# Transcriptomic responses to diet quality and viral infection in Apis mellifera

**DOI:** 10.1186/s12864-019-5767-1

**Published:** 2019-05-22

**Authors:** Lindsay Rutter, Jimena Carrillo-Tripp, Bryony C. Bonning, Dianne Cook, Amy L. Toth, Adam G. Dolezal

**Affiliations:** 1 Bioinformatics and Computational Biology Program, Iowa State University, Ames, 50011 IA USA; 2 Department of Microbiology, Center for Scientific Research and Higher Education of Ensenada, Ensenada, 22860 Baja California Mexico; 3 Department of Entomology and Nematology, University of Florida, Gainesville, 32611 FL USA; 4 Econometrics and Business Statistics, Monash University, Clayton, 3800 VIC Australia; 5 Department of Entomology, Iowa State University, Ames, 50011 IA USA; 6 Department of Ecology, Evolution, and Organismal Biology, Iowa State University, Ames, 50011 IA USA; 7 Department of Entomology, University of Illinois at Urbana-Champaign, Urbana, 61801 IL USA

**Keywords:** Honey bee, RNA-sequencing, Israeli acute paralysis virus, Monofloral pollen, Visualization

## Abstract

**Background:**

Parts of Europe and the United States have witnessed dramatic losses in commercially managed honey bees over the past decade to what is considered an unsustainable extent. The large-scale loss of bees has considerable implications for the agricultural economy because bees are one of the leading pollinators of numerous crops. Bee declines have been associated with several interactive factors. Recent studies suggest nutritional and pathogen stress can interactively contribute to bee physiological declines, but the molecular mechanisms underlying interactive effects remain unknown. In this study, we provide insight into this question by using RNA-sequencing to examine how monofloral diets and Israeli acute paralysis virus inoculation influence gene expression patterns in bees.

**Results:**

We found a considerable nutritional response, with almost 2000 transcripts changing with diet quality. The majority of these genes were over-represented for nutrient signaling (insulin resistance) and immune response (Notch signaling and JaK-STAT pathways). In our experimental conditions, the transcriptomic response to viral infection was fairly limited. We only found 43 transcripts to be differentially expressed, some with known immune functions (argonaute-2), transcriptional regulation, and muscle contraction. We created contrasts to explore whether protective mechanisms of good diet were due to direct effects on immune function (resistance) or indirect effects on energy availability (tolerance). A similar number of resistance and tolerance candidate differentially expressed genes were found, suggesting both processes may play significant roles in dietary buffering from pathogen infection.

**Conclusions:**

Through transcriptional contrasts and functional enrichment analysis, we contribute to our understanding of the mechanisms underlying feedbacks between nutrition and disease in bees. We also show that comparing results derived from combined analyses across multiple RNA-seq studies may allow researchers to identify transcriptomic patterns in bees that are concurrently less artificial and less noisy. This work underlines the merits of using data visualization techniques and multiple datasets to interpret RNA-sequencing studies.

**Electronic supplementary material:**

The online version of this article (10.1186/s12864-019-5767-1) contains supplementary material, which is available to authorized users.

## Background

Managed honey bees have undergone health declines in the United States and parts of Europe over the past decade [1–3], with annual mortality rates exceeding what beekeepers consider sustainable [4, 5]. More than 70 percent of major global food crops (including fruits, vegetables, and nuts) at least benefit from pollination, and yearly insect pollination services are valued worldwide at $175 billion [6]. As honey bees are largely considered to be the leading pollinator of numerous crops, their marked loss has considerable implications for agricultural sustainability [7].

Honey bee declines have been associated with several factors, including pesticide use, parasites, pathogens, habitat loss, and poor nutrition [8, 9]. Researchers generally agree that these stressors do not act in isolation; instead, they appear to influence the large-scale loss of honey bees in an interactive fashion as the environment changes [10]. Nutrition and viral infection are two factors that pose heightened dangers to honey bee health in response to recent environmental changes. Interactions between nutrition and viral infection may create feedbacks that impact bee health through several mechanisms [11, 12].

Pollen is a main source of nutrition (including proteins, amino acids, lipids, sterols, starch, vitamins, and minerals) in honey bees [13, 14]. At the individual level, pollen supplies most of the nutrients necessary for physiological development [15] and is believed to have considerable impact on longevity [16]. At the colony level, pollen enables young workers to produce jelly, which then nourishes larvae, drones, older workers, and the queen [17, 18]. Various environmental changes (including urbanization and monoculture crop production) have significantly altered the nutritional profile available to honey bees. In particular, honey bees are confronted with a less diverse selection of pollen, which is of concern because mixed-pollen (polyfloral) diets are generally considered healthier than single-pollen (monofloral) diets [19–21]. Reported colony mortality rates are higher in developed land areas compared to undeveloped land areas [22], and beekeepers rank poor nutrition as one of the main reasons for colony losses [23]. Understanding how low diversity diets (i.e. monofloral diets) affect honey bee health will be crucial to resolve problems that may arise as agriculture continues to intensify throughout the world [24, 25]. Indeed, differing qualities of monofloral diets have been shown to affect nurse bee physiology and tolerance to parasites [26].

Viral infection was considered a comparatively minor problem in honey bees until the last century when the ectoparasitic varroa mite (*Varroa destructor*) spread worldwide [27–29]. This mite feeds on honey bee hemolymph and/or fat body tissue [30, 31], and is believed to decrease lipid and glycogen reserves and reduce protein synthesis in bees [32]. Additionally, it transmits multiple viruses and supports replication of some viruses [33–36]. More than 20 honey bee viruses have been identified [37]. One of these viruses that has been linked to honey bee decline is Israeli acute paralysis virus (IAPV), a positive-sense RNA virus of the family *Dicistroviridae* [38]. IAPV infection causes shivering wings, decreased locomotion, muscle spasms, paralysis, and high premature death percentages in caged infected adult honey bees [39]. IAPV has demonstrated higher infectious capacity [40] and is more prevalent in colonies that do not survive the winter [41].

Although there is growing interest in how viruses and diet quality affect the health and sustainability of honey bees, as well as a recognition that such factors might operate interactively, there are only a small number of experimental studies thus far directed toward elucidating the interactive effects of these two factors in honey bees [42–46]. We recently used laboratory cages and nucleus hive experiments to investigate the health effects of these two factors, and our results show the importance of the combined effects of both diet quality and virus infection. Specifically, ingestion by honey bees of high quality pollen is able to mitigate virus-induced mortality to the level of diverse, polyfloral pollen [11].

Following up on these findings, we now aim to understand the corresponding underlying mechanisms by which high quality diets protect bees from virus-induced mortality. For example, it is not known whether the protective effect of good diet is due to direct, specific effects on immune function that reduce the pathogen load of the host (resistance, [47]) or if it is due to indirect effects of good nutrition on the ability of the host to withstand pathogen impacts without affecting pathogen load (tolerance, [47]). Transcriptomics is one means to better understand the mechanistic underpinnings of dietary and viral effects on honey bee health. Transcriptomic analysis can help us identify 1) the genomic scale of transcriptomic response to diet and virus infection, 2) whether these factors interact in an additive or synergistic way on transcriptome function, and 3) the types of pathways affected by diet quality and viral infection, which can help us generate candidate gene lists to further investigate the relative roles of tolerance and resistance. This information, heretofore lacking in the literature, can help us better understand how good nutrition may be able to serve as a “buffer” against other stressors [12].

There are only a small number of published experiments examining gene expression patterns related to diet effects [48] and virus infection effects [49–53] in honey bees, but there have been several such studies in model organisms. Model insect studies can inform studies of honey bee transcriptomic responses, using functional inference of as-of-yet uncharacterized honey bee genes based on orthology to Drosophila and other model organisms. Previous Drosophila studies that examined various diet effects have found gene expression changes related to immunity, metabolism, cell cycle activity, DNA binding, transcription, and insulin signaling [48, 54–56]. While similar transcriptomic studies have been limited in honey bees, one study found that pollen nutrition upregulates genes involved in macromolecule metabolism, longevity, and the insulin/TOR pathway [48, 51]. Previous transcriptomic studies have identified genes serving as links between metabolism and antiviral defense in honey bees [57, 58]; see [59] for an overview. Numerous studies on the transcriptomic effects of virus infection in model insect organisms have shown that RNA silencing, transcriptional pausing, Toll pathways, IMD pathways, JAK/STAT pathways, and Toll-7 autophagy pathways play substantial roles in virus-host systems [60, 61]. Studies of virus-bee systems have revealed some of the antiviral defense pathways known in model organisms are conserved and also related to bee antiviral immune responses [62].

To our knowledge, there are few to no studies investigating honey bee gene expression patterns specifically related to monofloral diets, and few studies investigating honey bee gene expression patterns related to the combined effects of diet in any broad sense and viral inoculation in any broad sense [45]. In this study, we examine how monofloral diets and viral inoculation influence gene expression patterns in honey bees by focusing on four treatment groups (low quality diet without IAPV inoculation, high quality diet without IAPV inoculation, low quality diet with IAPV inoculation, and high quality diet with IAPV inoculation). For our diet factor, we examined two monofloral pollen diets, rockrose (*Cistus* sp.) and chestnut (*Castanea* sp.). Rockrose pollen is generally considered less nutritious than chestnut pollen because it contains smaller amounts of protein, amino acids, antioxidants, calcium, and iron [11, 26]. For specific quantitative differences between these two pollen groups, please see [26]. Throughout this paper, we refer to our four treatment groups as “NR” (non-inoculation and low quality rockrose pollen), “NC” (non-inoculation and high quality chestnut pollen), “VR” (IAPV inoculation and low quality rockrose pollen), and “VC” (IAPV inoculation and high quality chestnut pollen). We conduct RNA-sequencing analysis on a randomly selected subset of the honey bees we used in our previous study (as is further described in our methods section). We then examine pairwise combinations of treatment groups, the main effect of monofloral diet, the main effect of IAPV exposure, and the combined effect of the two factors on gene expression patterns.

Because RNA-seq data can be noisy and subject to high levels of inter-experiment variation, we further sought to validate our transcriptomic data via comparison to a previous RNA-seq study on honey bee responses to viral infection. To do this, we compare the main effect of IAPV exposure in our dataset to that obtained in a previous study conducted by Galbraith and colleagues [49]. While our study examines honey bees derived from naturally-mated queens, the Galbraith study examined honey bees derived from single-drone inseminated queens. As a consequence, intracolony worker relatedness in our study is around 25%, compared to 75% in the Galbraith study [63]. Genetic diversity of bees in our study was thus higher than the Galbraith study, and was likely to be especially high because we sampled from 15 different colonies, i.e. from 15 different, naturally-mated queens. We should therefore expect that the Galbraith study may generate data with higher signal-to-noise ratios than our data due to lower genetic variation between its replicates. At the same time, our honey bees will be more likely to display the health benefits gained from increased genotypic variance within colonies, including decreased parasitic load [64], increased tolerance to environmental changes [65], and increased colony performance [66, 67]. Given that honey bees are naturally very polyandrous [68], our naturally-mated honey bees may also reflect more realistic environmental and genetic conditions. To achieve this comparison, we use visualization techniques to assess the signal:to:noise ratio between these two datasets, and differential gene expression (DEG) analyses to determine any significantly overlapping genes of interest between these two datasets. As RNA-sequencing data can be biased [69–71], this comparison allowed us to characterize how repeatable and robust our RNA-sequencing results were in comparison to previous studies. It also allowed us to shine light on how experimental designs that control genetic variability to different extents might affect the resulting gene expression data in honey bees. We suggest that in-depth data visualization approaches (including scatterplot matrices, parallel coordinate plots, and litre plots from the bigPint software package [72]) can be useful for cross-study comparisons and validation of noisy RNA-sequencing data in the future.

## Methods

### Mortality and virus titers

Details of the procedures we used to prepare virus inoculum, infect and feed caged honey bees, and quantify IAPV can be reviewed in our previous work [11, 40]. In brief, our virus inoculum was prepared by injection of infectious virus particles (derived from infected adults) into white-eyed honey bee pupae; these pupae were then homogenized and virus particles enriched and resuspended. This inoculum was then characterized for presence of acute bee paralysis virus, black queen cell virus, deformed wing virus (DWV), IAPV, Kashmir bee virus, and sacbrood bee virus (SBV). Experimental infection tests of adult bees and honey bee cell cultures [40] showed that only IAPV is amplified in adult bees. To infect caged bees for these experiments, newly emerged bees from 15 healthy colonies at the Iowa State University research apiary were homogeneously mixed, then counted into clear acrylic cages in groups of 35 bees per cage. Cages were then presented with open feeders containing 30% sucrose solution (control) or 30% sucrose solution containing a 1:1000 dilution of viral inoculum (treatment). Dietary treatments were then added (described below). To quantify virus titers, two live bees were randomly sampled at 36 hpi from each of 9–10 randomly selected cages. Virus levels were then measured via RT-qPCR and quantified against a standard curve, identically to methods described in [11, 40].

A linear mixed effects model was used to relate the mortality rates and IAPV titers to the main and interaction effects of the diet and virus factors. The model was fit to the data by restricted maximum likelihood (REML) using the “lme” function in the R package “nlme”. A random (intercept) effect for experimental setup was included in the model. Post-hoc pairwise comparisons of the four (diet and virus combination) treatment groups were performed and Benjamini-Hochberg adjusted *p*-values were calculated to limit familywise Type I error rates [73].

### Design of two-factor experiment

For our nutrition factor, we examined two monofloral pollen diets, rockrose (*Cistus* sp.) and chestnut (*Castanea* sp.). Rockrose pollen is generally considered less nutritious than chestnut pollen due to its lower levels of protein, amino acids, antioxidants, calcium, and iron [11, 26]. For our virus factor, one level contained bees that were inoculated with IAPV and another level contained bees that were not inoculated with IAPV. This experimental design resulted in four treatment groups (NR: low quality rockrose pollen without IAPV exposure; NC: high quality chestnut pollen without IAPV exposure; VR: low quality rockrose pollen with IAPV exposure; VC: high quality chestnut pollen with IAPV exposure) that allowed us to assess main effects and interactive effects between diet quality and IAPV infection in honey bees.

There are several reasons why our design focused only on diet quality (monofloral diets) as opposed to diet diversity (monofloral diets versus polyfloral diets). First, when assessing diet diversity, a sugar diet is often used as a control. However, such an experimental design does not reflect real-world conditions for honey bees as they rarely face a total lack of pollen [26]. Moreover, younger larvae tend to be fed pollen diets, whereas older larvae tend to be fed nectar diets. By focusing on pollen diets, our study design reflects natural diet conditions for larvae of a specific age category [74]. Second, in studies that compared honey bee health using monofloral and polyfloral diets at the same time, if the polyfloral diet and one of the high quality monofloral diets both exhibited similarly beneficial effects, then it was difficult for the authors to assess if the polyfloral diet was better than most of the monofloral diets because of its diversity or because it contained as a subset the high quality monofloral diet [26]. Third, as was previously mentioned, honey bees are now confronted with less diverse sources of pollen. As a result, there is a need to better understand how monofloral diets affect honey bee health.

### RNA extraction

Fifteen cages per treatment were originally produced for monitoring of mortality. From these, six live honey bees were randomly selected from each cage 36 h post inoculation and placed into tubes [40]. In summary, 8 samples (representing two bees each) were sequenced per experimental condition (i.e., 32 samples sequenced). Tubes were kept on dry ice and then transferred into a -80C freezer until processing. From the fifteen possible cages, eight were randomly selected for RNA-sequencing. From these eight cages, two of the honey bees per cage were randomly selected from the original six live honey bees per cage. These two bees were combined to form a pooled sample representing the cage. Whole body RNA from each pool was extracted using Qiagen RNeasy MiniKit followed by Qiagen DNase treatment. Samples were suspended in water to 200-400 ng/ *μ*l. All samples were then tested on a Bioanalyzer at the Iowa State University DNA Facility to ensure quality (RIN >8).

### Gene expression

Samples were sequenced starting on January 14, 2016 at the Iowa State University DNA Facility (Platform: Illumina HiSeq Sequencing 2500 in rapid run mode; Category: Single End 100 cycle sequencing). A standard Illumina mRNA library was prepared by the DNA facility. Reads were aligned to the BeeBase Version 3.2 genome [75] from the Hymenoptera Genome Database [76] using the programs GMAP and GSNAP [77]. There were four lanes of sequencing with 24 samples per lane. Each sample was run twice. Approximately 75-90% of reads were mapped to the honey bee genome. Each lane produced around 13 million single-end 100 basepair reads.

We tested all six pairwise combinations of treatments for DEGs (pairwise DEGs: NR versus NC, NR versus VR, NR versus VC, NC versus VR, NC versus VC, and VR versus VC). We also tested the diet main effect (diet DEGs), virus main effect (virus DEGs), and interaction term for DEGs (interaction DEGs). We then also tested for virus main effect DEGs (virus DEGs) in public data derived from a previous study exploring the gene expression of IAPV virus infection in honey bees [49]. We tested each DEG analysis using recommended parameters with DESeq2 [78], edgeR [79], and LimmaVoom [80]. For our DEG analysis, we used R software version 3.3.3 [81]. In all cases, we used a false discovery rate (FDR) threshold of 0.05 [82]. Fisher’s exact test was used to determine significant overlaps between DEG sets (whether from the same dataset but across different analysis pipelines or from different datasets across the same analysis pipelines). The eulerr shiny application was used to construct Venn diagram overlap images [83]. In the end, we focused on the DEG results from DESeq2 [78] as this pipeline was also used in the Galbraith study [49]. We used the independent filtering process built into the DESeq2 software that mitigates multiple comparison corrections on genes with no power rather than defining one filtering threshold.

### Comparison to prior studies on transcriptomic response to viral infection

We compare the main effect of IAPV exposure in our dataset to that obtained in a previous study conducted by Galbraith and colleagues [49] who also addressed honey bee transcriptomic responses to virus infection. We applied the same downstream bioinformatics analyses between our count table and the count table provided in the Galbraith study. When we applied our bioinformatics pipeline to the Galbraith count table, we obtained different differential expression counts compared to the results published in the Galbraith study. However, there was substantial overlap and we considered this justification to use the differential expression list we obtained in order to keep the downstream bioinformatics analyses as similar as possible between the two datasets (Additional file 17).

We used honey bees from naturally-mated colonies, whereas Galbraith et al. [49] used honey bees from single-drone colonies. In light of this, we should expect the Galbraith et al. dataset to contain lower genetic variation between its replicates and higher signal-to-noise ratios than our dataset. We use visualization techniques to assess the signal-to-noise ratio between these two datasets, and differential gene expression (DEG) analyses to determine any significantly overlapping genes of interest between these two datasets.

### Visualization

We used an array of visualization tools as part of our analysis. We used the PCA plot [84] from the DESeq2 package, a well-known and established tool. Along with that, we used lesser-known multivariate visualization tools from our R package called bigPint [72]. Specifically, we used parallel coordinate plots [85], scatterplot matrices [86], and litre plots (which we recently developed based on “replicate line plots” [87]) to assess the variability between the replicates and the treatments in our data. We also used these plotting techniques to assess for normalization problems and other common problems in RNA-sequencing analysis pipelines [87].

Furthermore, we used statistical graphics to better understand patterns in our DEGs. However, in cases of large DEG lists, these visualization tools had overplotting problems (where multiple objects are drawn on top of one another, making it impossible to detect individual values). To remedy this problem, we first standardized each DEG to have a mean of zero and standard deviation of unity for its read counts across its samples [88, 89]. Then, we performed hierarchical clustering on the standardized DEGs using Ward’s linkage. This process divided large DEG lists into smaller clusters of similar patterns, which allowed us to more efficiently visualize the different types of patterns within large DEG lists (see Figs. 3 and 4 for examples).

**Fig. 1:**
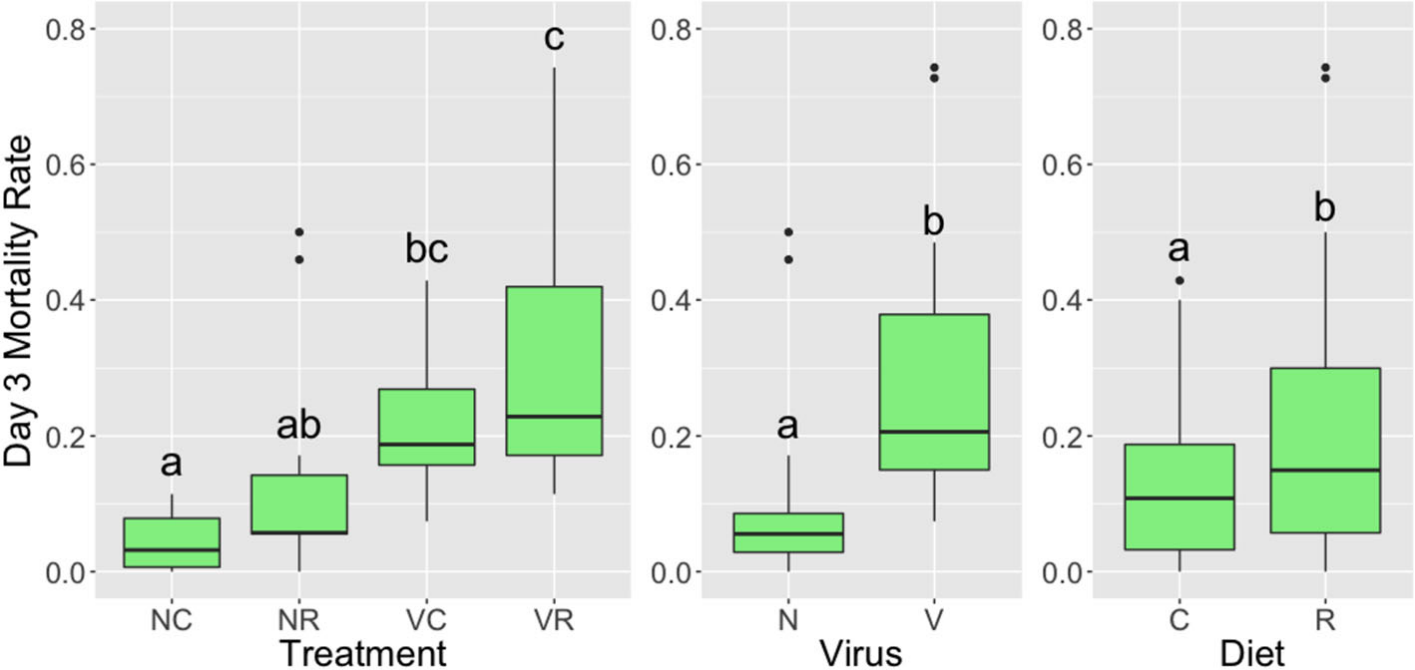
Mortality rates for the four treatment groups, two virus groups, and two diet groups. Left to right: Mortality rates for the four treatment groups, two virus groups, and two diet groups. “N” represents non-inoculation, “V” represents viral inoculation, “C” represents chestnut pollen, and “R” represents rockrose pollen. The mortality rate data included 59 samples with 15 replicates per treatment group, except for the “NC” group having 14 replicates. ANOVA values and *p*-values for the statistical tests are listed in the text of the paper. The letters above the bars represent significant differences with a confidence level of 95%

**Fig. 2:**
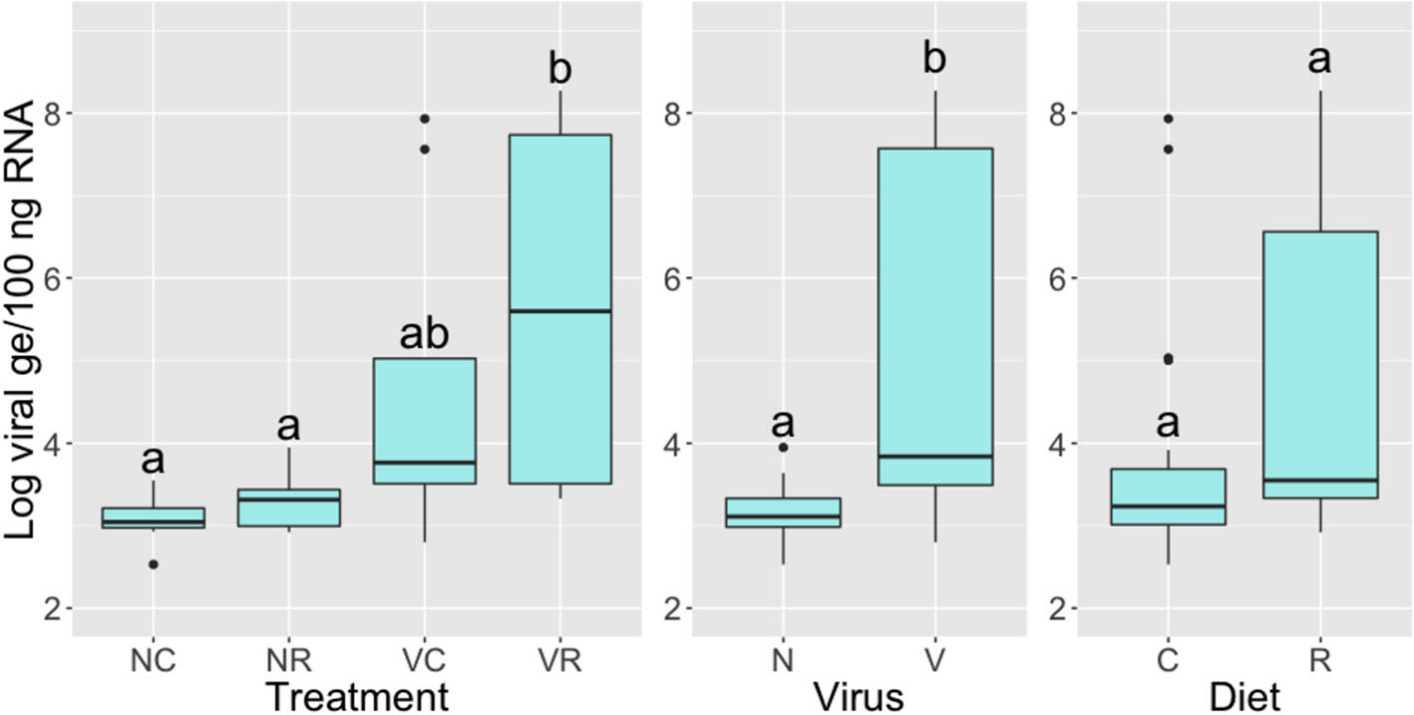
IAPV titers for the four treatment groups, two virus groups, and two diet groups. Left to right: IAPV titers for the four treatment groups, two virus groups, and two diet groups. “N” represents non-inoculation, “V” represents viral inoculation, “C” represents chestnut pollen, and “R” represents rockrose pollen. The IAPV titer data included 38 samples with 10 replicates per treatment group, except for the “NR” group having 8 replicates. ANOVA values and *p*-values for the statistical tests are listed in the text of the paper. The letters above the bars represent significant differences with a confidence level of 95%

**Fig. 3:**
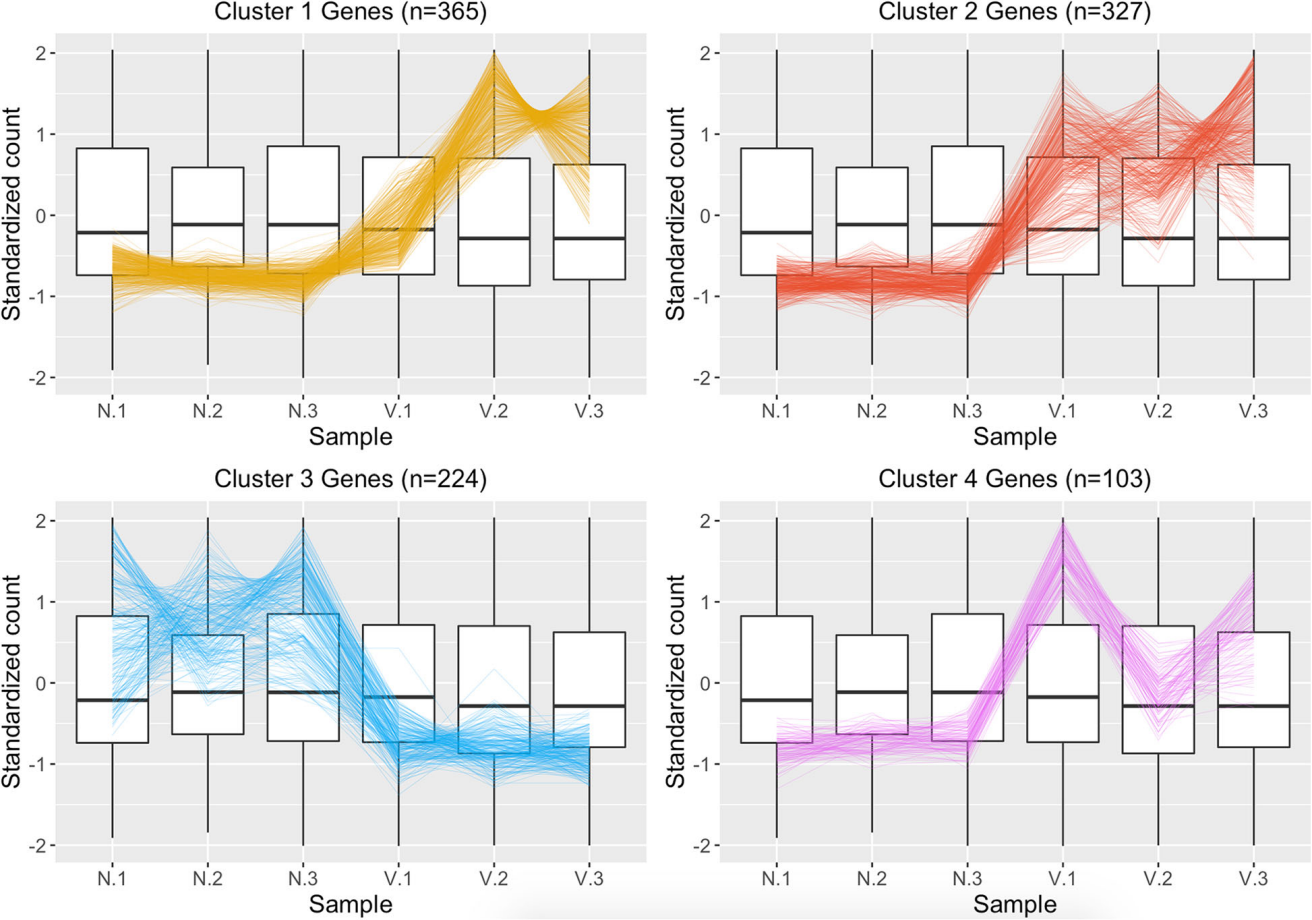
Parallel coordinate plots of the 1019 virus-related DEGs of the Galbraith data [49]. Parallel coordinate plots of the 1019 DEGs after hierarchical clustering of size four between the virus-infected and control groups of the Galbraith study. “N” represents non-inoculation, “V” represents viral inoculation. Clusters 1, 2, and 4 seem to represent DEGs that were overexpressed in the virus inoculated group, and Cluster 3 seems to represent DEGs that were overexpressed in the non-inoculated control group. In general, the DEGs appeared as expected, but there is rather noticeable deviation of the first replicate from the virus-treated sample (“V.1”) from the other virus-treated replicates in Cluster 1. We also note a deviation of the second replicate from the virus-treated samples (“V.2”) from the other virus-treated replicates in Cluster 4

**Fig. 4:**
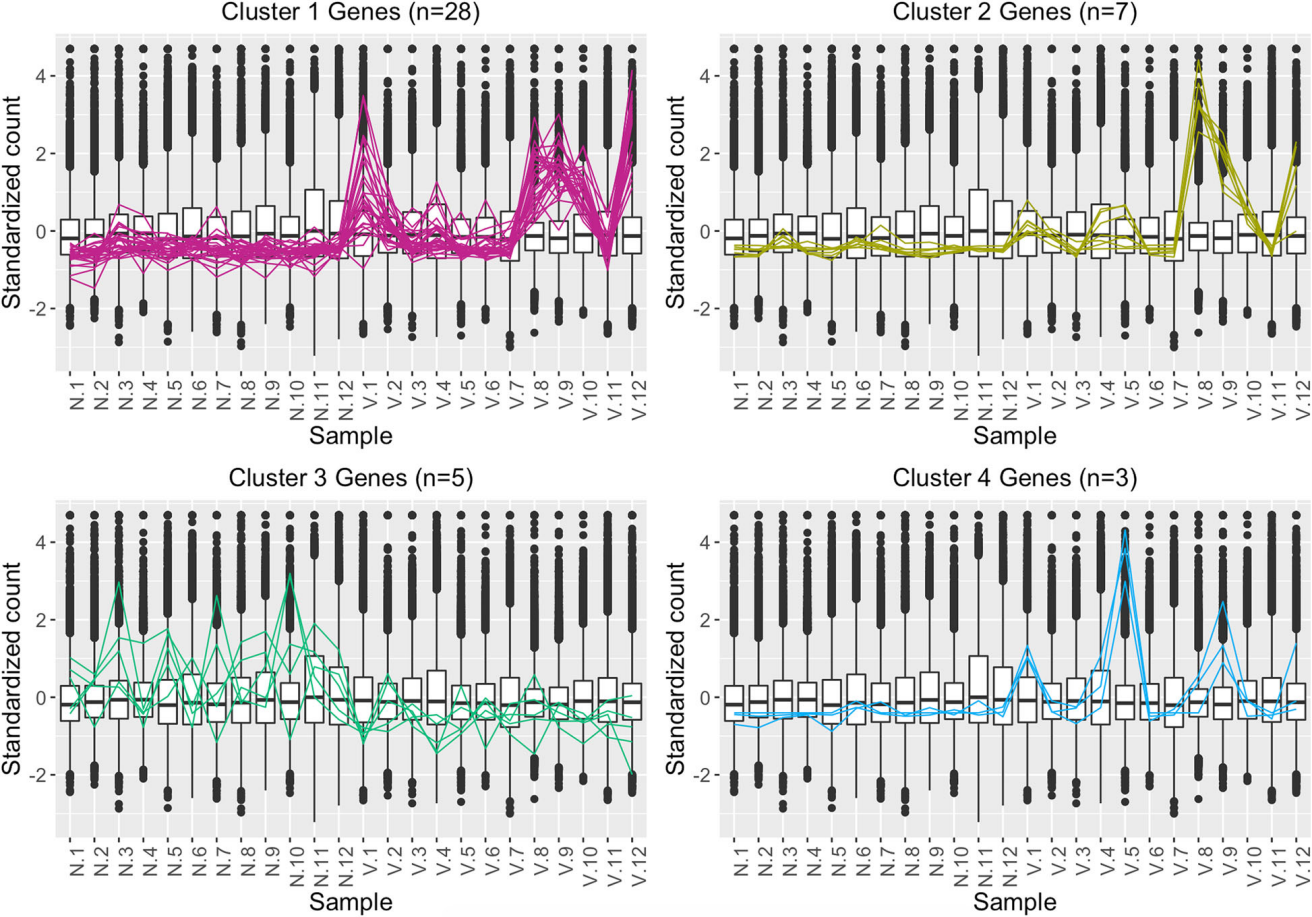
Parallel coordinate plots of the 43 virus-related DEGs of our data. Parallel coordinate plots of the 43 DEGs after hierarchical clustering of size four between the virus-inoculated and control groups of our study. “N” represents non-inoculated control group, and “V” represents treatment of virus. The vertical red line indicates the distinction between treatment groups. We see from this plot that the DEG designations for this dataset do not appear as clean compared to what we saw in the Galbraith dataset in Fig. 3

### Gene ontology

DEGs were uploaded as a background list to DAVID Bioinformatics Resources 6.7 [90, 91]. The overrepresented gene ontology (GO) terms of DEGs were determined using the BEEBASE_ID identifier option (honey bee gene model) in the DAVID software. To fine-tune the GO term list, only terms correlating to Biological Processes were considered. The refined GO term list was then imported into REVIGO [92], which uses semantic similarity measures to cluster long lists of GO terms.

### Probing tolerance versus resistance

To investigate whether the protective effect of good diet is due to direct, specific effects on immune function (resistance), or if it is due to indirect effects of good nutrition on energy availability and vigor (tolerance), we created contrasts of interest (Table 2). In particular, we assigned “resistance candidate DEGs” to be the ones that were upregulated in the chestnut group within the virus inoculated bees but not upregulated in the chestnut group within the non-inoculated bees. Our interpretation of these genes is that they represent those that are only activated in inoculated bees that are fed a high quality diet. We also assigned “tolerance candidate DEGs” to be the ones that were upregulated in the chestnut group for both the virus inoculated bees and non-inoculated bees. Our interpretation of these genes is that they represent those that are constitutively activated in bees fed a high quality diet, regardless of whether they are experiencing infection or not. We then determined how many genes fell into these two categories and analyzed their GO functions.

**Table 1:** Known functions of the mapped subset of 43 DEGs in the virus main effect of our study

BeeBase ID, NCBI Gene ID	Gene Name	Known functions	Us	Galbraith
GB41545, 409187	MD-2-related lipid-recognition protein-like	Implicated in lipid recognition, particularly in the recognition of pathogen related products	N	-
GB50955, 411577	Protein argonaute-2	Interacts with small interfering RNAs to form RNA-induced silencing complexes which target and cleave transcripts that are mostly from viruses and transposons	V	V
GB48755, 727455	UBA-like domain-containing protein 2	Found in diverse proteins involved in ubiquitin/proteasome pathways	V	V
GB47407, 406132	Histone H4	Capable of affecting transcription, DNA repair, and DNA replication when post-transcriptionally modified	V	V
GB42313, 409923	Leishmanolysin-like peptidase	Encodes a protein involved in cell migration and invasion; implicated in mitotic progression in D. melanogaster	V	V
GB50813, 410127	Rho guanine nucleotide exchange factor 11	Implicated in regulation of apoptopic processes, cell growth, signal transduction, and transcription	V	V
GB54503, 411255	Thioredoxin domain-containing protein	Serves as a general protein disulphide oxidoreductase	N	-
GB53500, 100576392	Transcriptional regulator Myc-B	Regulator gene that codes for a transcription factor	V	V
GB51305, 551252	Tropomyosin-like	Related to protein involved in muscle contraction	N	N
GB50178, 726905	Cilia and flagella-associated protein 61-like	Induces components required for wild-type motility and stable assembly of motile cilia	V	V

Whether the gene was overrepresented in the virus or non-virus group is also indicated for both our study and the Galbraith study. Functionalities were extracted from Flybase, National Center for Biotechnology Information and The European Bioinformatics Institute databases

**Table 2:** Contrasts in our study for assessing GO and pathways analysis

Contrast	DEGs	Interpretation	Results
V (all) vs N (all)	43	Genes that change expression due to virus effect regardless of diet status in bees	Table 1
NC vs NR	941	Genes that change expression due to diet effect in non-inoculated bees	Additional file 1: Table S4 and 5
VC vs VR	376	Genes that change expression due to diet effect in inoculated bees	Additional file 1: Table S6 and 7
VC upregulated in VC vs VR, and NC upregulated in NC vs NR	122	“Tolerance” genes that turn on by good diet regardless of virus infection status in bees	Fig. 5a
VC upregulated in VC vs VR, but NC not upregulated in NC vs NR	125	“Resistance” genes that turn on by good diet only in inoculated bees	Fig. 5b

### Post hoc analysis

We found considerable noisiness in our data and saw, through gene-level visualizations, that our DEGs contained outliers and inconsistent replicates. Hence, we wanted to explore whether our DEG read counts correlated with pathogen response metrics, including IAPV titers, SBV titers (also present in our inoculum [11, 40]), and mortality rates. We explored correlation with SBV because our inoculum [40] does contain SBV, and bees from both inoculated and non-inoculated groups do exhibit detectable SBV titers. For this process, we considered virus main effect DEGs (Fig. 4), “tolerance candidate” DEGs (Additional file 15), and “resistance candidate” DEGs (Additional file 16). For each DEG in each cluster, we calculated a coefficient of determination (R-squared) value to estimate the correlation between its raw read counts and the pathogen response metrics across its 24 samples. We then used the Kruskal–Wallis test to determine if the distribution of the R-squared values in any of the DEG clusters significantly differed from those in the non-DEG genes (the rest of the data). As there were four clusters for each of the nine combinations of DEG lists (“tolerance” candidate DEGs, “resistance” candidate DEGs, and virus-related DEGs) and pathogen response measurements (IAPV titer, SBV titer, and mortality rate), this process resulted in 36 statistical tests.

## Results

### Mortality and virus titers

We reanalyzed our previously published dataset with a subset that focuses on diet quality and is more relevant to the current study. We show the data subset here to inform the RNA-sequencing comparison because we reduced the number of treatments from the original published data (from eight to four) [11] as a means to focus on diet quality effects.

As shown in Fig. 1, mortality rates of honey bees 72 h post-inoculation significantly differed among the treatment groups (mixed model ANOVA across all treatment groups, df = 3, 54; F = 10.03; p <2.34e-05). The effect of virus treatment (mixed model ANOVA, df = 1, 54; F = 24.73; p <7.04e-06) and diet treatment (mixed model ANOVA, df = 1, 54; F = 5.32; p <2.49e-02) were significant, but the interaction between the two factors (mixed model ANOVA, df = 1, 54; F = 4.72e-02, p = 8.29e-01) was not significant. We compared mortality levels based on pairwise comparisons: For a given diet, honey bees exposed to the virus showed significantly higher mortality rate than honey bees not exposed to the virus. Bees fed rockrose pollen had significantly elevated mortality with virus infection compared to non-inoculated controls (Benjamini-Hochberg, p <1.53e-03), and bees fed chestnut pollen similarly had significantly elevated mortality with virus infection compared to controls (Benjamini-Hochberg, p <3.12e-03) (Fig. 1).

As shown in Fig. 2, IAPV titers of honey bees 72 h post-inoculation significantly differed among the treatment groups (mixed model ANOVA across all treatment groups, df = 3, 33; F = 6.10; p <2.03e-03). The effect of virus treatment (mixed model ANOVA, df = 1, 33; F = 15.04; p <4.75e-04) was significant, but the diet treatment (mixed model ANOVA, df = 1, 33; F = 2.55; p = 1.20e-01) and the interaction between the two factors (mixed model ANOVA, df = 1, 33; F = 7.02e-01, p = 4.08e-01) were not significant. We compared IAPV titers based on pairwise comparisons: Bees fed rockrose pollen had significantly elevated IAPV titers with virus infection compared to non-inoculated controls (Benjamini Hochberg, p <7.56e-03). However, bees fed chestnut pollen did not have significantly elevated IAPV titers with virus infection compared to non-inoculated controls (Benjamini Hochberg, p = 6.29e-02). While many of the non-inoculated treatment groups showed some RT-qPCR amplification (non-inoculated average Ct=33.92; inoculated average Ct=24.9), and thus have virus titers calculable on a standard curve, these Ct levels are similar to those deemed uninfected in previous studies [49]. Overall, we interpreted these findings to mean that high quality chestnut pollen could partially reduce high virus titers resulting from the inoculation treatment, whereas low quality rockrose pollen could not (Fig. 2).

### Transcriptomic responses to virus infection and diet

In bees collected 36 h post treatment, we observed a substantially larger number of differentially expressed genes (DEGs) in our diet main effect (*n* = 1914) than in our virus main effect (*n* = 43) (Additional file 1: Table S1A and B). There were only four genes that were DEGs in both our diet main effect and our virus main effect (GB48747, GB47214, GB42908, and GB42507). In the diet factor, more DEGs were upregulated in the more-nutritious chestnut group (*n* = 1033) than in the less-nutritious rockrose group (*n* = 881). In the virus factor, there were more virus-upregulated DEGs (*n* = 38) than control-upregulated DEGs (*n* = 5). While these reported DEG counts are from the DESeq2 package, we saw similar trends for the edgeR and limma package results (Additional file 1: Table S1 and Additional file 18).

We performed GO analysis to statistically assign our DEGs to predefined bins based on their functional characteristics, allowing us to better understand the biological processes of our DEGs. GO analysis of the chestnut-upregulated DEGs revealed the following over-represented biological functions: Wnt signaling, hippo signaling, and dorso-ventral axis formation, as well as pathways related to circadian rhythm, mRNA surveillance, insulin resistance, inositol phosphate metabolism, FoxO signaling, ECM-receptor interaction, phototransduction, Notch signaling, JaK-STAT signaling, MAPK signaling, and carbon metabolism (Additional file 1: Table S2). These encompassed almost all of the overrepresented biological functions in chestnut-upregulated DEGs conditioned on non-inoculation (i.e. upregulated in the “NC” group compared to the “NR” group; Additional file 1: Table S4) and inoculation (i.e. upregulated in the “VC” group compared to the “VR” group; Additional file 1: Table S6). GO analysis of the rockrose DEGs revealed pathways related to terpenoid backbone biosynthesis, homologous recombination, SNARE interactions in vesicular transport, aminoacyl-tRNA biosynthesis, Fanconi anemia, and pyrimidine metabolism (Additional file 1: Table S3). We note that Fanconi anemia pathways was also the only GO term discovered in rockrose DEGs conditioned on viral inoculation (i.e. upregulated in the “VR” group compared to the “VC” group) (Additional file 1: Table S7). However, Fanconi anemia pathways were not found in rockrose DEGs conditioned on non-inoculation (i.e. upregulated in the “NR” group compared to the “NC” group) (Additional file 1: Table S5).

With so few DEGs (*n* = 43) in our virus main effect comparison, we focused on individual genes and their known functionalities rather than GO over-representation (Table 1). Of the 43 virus-related DEGs, only 10 had GO assignments within the DAVID database. These genes had putative roles in the recognition of pathogen-related lipid products and the cleaving of transcripts from viruses, as well as involvement in ubiquitin and proteosome pathways, transcription pathways, apoptotic pathways, oxidoreductase processes, and several more functions (Table 1).

No interaction DEGs were observed between the diet and virus factors of the study, in any of the pipelines (DESeq2, edgeR, and limma).

The number of DEGs across the six treatment pairings between the diet and virus factor ranged from 0 to 955 (Additional file 1: Table S8). Again, diet level appeared to have greater influence on the number of DEGs than the virus level. Across every pair comparing the chestnut and rockrose levels, regardless of the virus level, the number of chestnut-upregulated DEGs was higher than the number of rockrose-upregulated DEGs (Additional file 1: Table S8C–F). Virus-treated bees showed equal to or more upregulated genes relative to controls, under both diet treatments (Additional file 1: Table S8A and B). These trends were observed for all three pipelines used (DESeq2, edgeR, and limma).

Additional file 20: Tables S1–9 contain complete DEG lists for all comparisons performed in this study.

### Transcriptomic data visualization and comparison to a previous study

We wished to explore the signal:to:noise ratio between the Galbraith dataset and our dataset. Note that the Galbraith dataset contained three individual bees per treatment group as a single pooled sample, while our dataset contained 16 individual bees per treatment group in 8 RNA-seq samples. Basic PCA plots were constructed with the DESeq2 analysis pipeline and showed that the Galbraith dataset may separate the inoculated and non-inoculated honey bees better than our dataset (Additional file 2). Wanting to learn more about the data at the gene level, we continued with new visualization techniques that are available online [72]. For more information about the visualizations used here, please refer to (https://lindsayrutter.github.io/bigPint/articles/plotIntro.html).

We used parallel coordinate lines superimposed onto side-by-side boxplots to visualize the DEGs associated with virus infection in the two studies. The background side-by-side boxplot represents the distribution of *all* genes in the data (all 15,314 genes in our count table), and each parallel coordinate line represents one DEG. In a parallel coordinate line, connections between samples with positive correlations should be flat, while connections between samples with negative correlations should be crossed. We expect DEGs to show more variability between treatments than between replicates. This means the parallel coordinate lines should be flat between replicates but crossed between treatments. However, overplotting problems would obscure our visualization if we were to plot all DEGs onto the same side-by-side boxplot. Therefore, we graphed clustered subsets of the DEGs (based on hierarchical clustering).

The 1019 DEGs from the Galbraith dataset form relatively clean-looking visual displays, with consistent replicates and differences between treatments. The few inconsistent replicates we observed (such as V.1 from Cluster 1 and V.2 from Cluster 4) were small enough that consistent differences between the treatment groups remained apparent across the samples (Fig. 3). In contrast, we see that the 43 virus-related DEGs from our dataset do not look as clean in their visual displays (Fig. 4). The replicates appear somewhat inconsistent in their estimated expression levels and there is not always such a large (or even consistent) difference between treatment groups. We see a similar finding when we also examine a larger subset of 1914 diet-related DEGs from our study (Additional file 3).

We next used repLIcate TREatment (“litre”) plots, which we recently developed for our bigPint software package [72]. Litre plots allow users to visualize one DEG onto the Cartesian coordinates of one scatterplot matrix. In the litre plot, each gene in the data is plotted once for every combination of replicates between treatment groups. We use hexagon bins to summarize this massive information. Once the background of hexagons has been drawn to reveal the distribution of all between-treatment sample pair combinations for *all* genes, the user can superimpose all between-treatment sample pair combinations for one gene of interest.

Additional file 4 shows nine example litre plots for our dataset; each litre plot shows the 144 between-treatment sample pair combinations for one DEG of interest. Additional files 5 and 6 similarly each show nine example litre plots for the Galbraith dataset; each litre plot shows the nine between-treatment sample pair combinations for one DEG of interest. We see that indeed the virus DEGs from our data (Additional file 4) show less consistent replications and less differences between the treatment groups compared to the virus DEGs from the Galbraith data (Additional files 5 and 6). We also observe that, in the Galbraith dataset, the DEG points in the first cluster show less tight cluster patterns than the DEG points in the second cluster (Additional files 5 and 6), an observation we saw previously in the parallel coordinate plots (Fig. 3).

Finally, we used scatterplot matrices from the bigPint software to further assess the DEGs [72]. A scatterplot matrix is another effective multivariate visualization tool that plots read count distributions across all genes and samples. Specifically, it represents every gene in the dataset as a black point in each scatterplot. DEGs can be superimposed as colored points to assess their patterns against the full dataset. We expect DEGs to mostly fall along the *x=y* line in replicate scatterplots (denoting replicate consistency) but deviate from the *x=y* line in treatment scatterplots (denoting significant treatment changes). The *x=y* line is shown in red in our plots.

We created standardized scatterplot matrices for each of the four clusters (from Fig. 3) of the Galbraith data (Additional files 7, 8, 9, and 10). We also created standardized scatterplot matrices for our data. However, as our dataset contained 24 samples, we would need to include 276 scatterplots in our matrix, which would be too numerous to allow for efficient visual assessment of the data. As a result, we created four scatterplot matrices of our data, each with subsets of 6 samples to be more comparable to the Galbraith data (Additional files 11, 12, 13, and 14). Specifically, we arbitrarily subsetted the samples so each one was represented once in each of these four files (i.e. Additional file 11 shows samples 1–3; Additional file 12 shows samples 4–6; Additional file 13 shows samples 7–9; and Additional file 14 shows samples 10–12). We can again confirm through these plots that the DEGs from the Galbraith data appeared more as expected: They deviated more from the *x=y* line in the treatment scatterplots while staying close to the *x=y* line in replicate scatterplots.

Despite the virus-related DEGs (*n* = 1019) from the Galbraith dataset displaying the expected patterns more than those from our dataset (*n* = 43), there was significant overlap (*p*-value <2.2e-16) in the DEGs between the two studies, with 26/38 (68%) of virus-upregulated DEGs from our study also showing virus-upregulated response in the Galbraith study (Fig. 6).

**Fig. 5:**
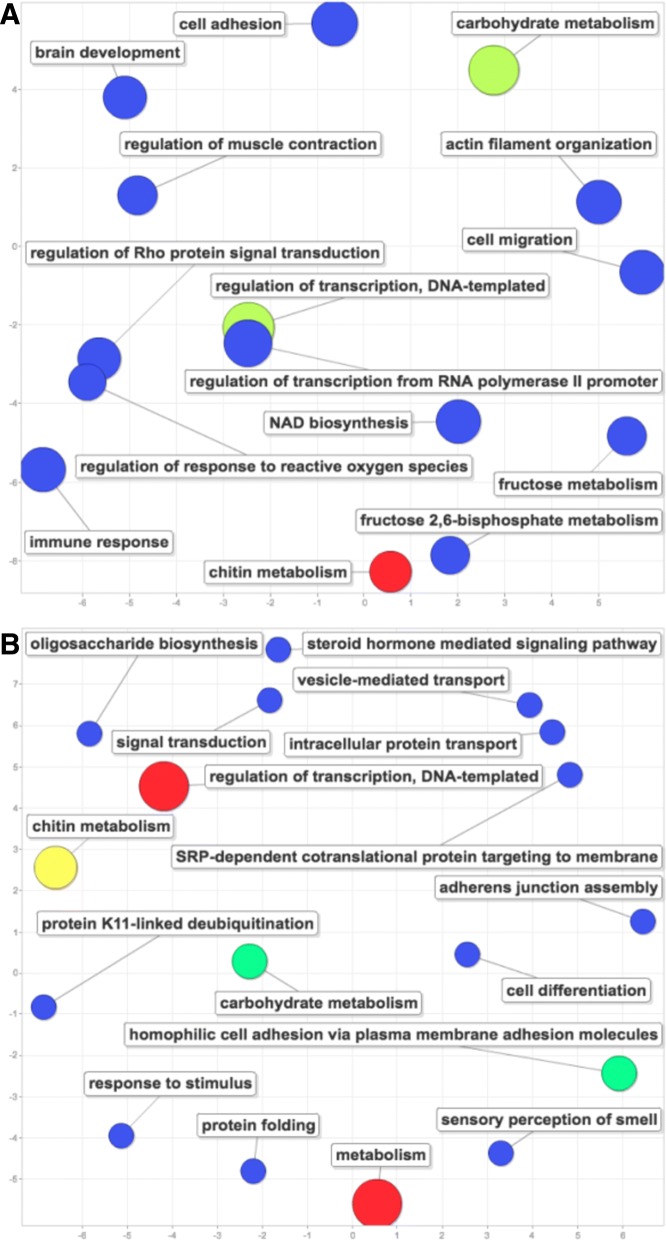
Gene ontology results for the 122 “tolerance” and 125 “resistance” DEG candidates in our data. GO analysis results for the 122 DEGs related to our “tolerance” hypothesis (A) and for the 125 DEGs related to our “resistance” hypothesis (B). The color and size of the circles both represent the number of genes in that ontology. The x-axis and y-axis are organized by SimRel, a semantic similarity metric [106]

**Fig. 6:**
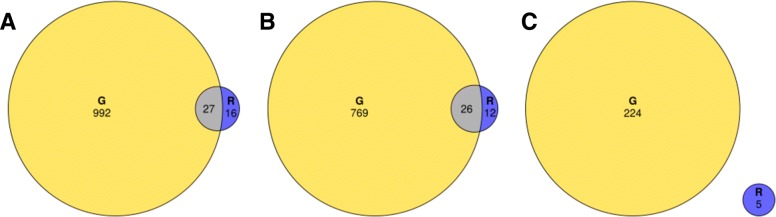
Venn diagrams comparing the virus-related DEG overlaps between our dataset and the Galbraith dataset. Venn diagrams comparing the virus-related DEG overlaps between the Galbraith study (labeled as “G”) and our study (labeled as “R”). From left to right: Total virus-related DEGs (subplot A), virus-upregulated DEGs (subplot B), control-upregulated DEGs (subplot C). Both the total virus-related and virus-upregulated DEGs showed significant overlap between the studies (*p*-value <2.2e-16) as per Fisher’s Exact Test for Count Data. There was one gene that was virus-upregulated in the Galbraith study but control-upregulated in our study

### Tolerance versus resistance

Using the contrasts specified in Table 2, we discovered 122 “tolerance” candidate DEGs and 125 “resistance” candidate DEGs. Within our 122 “tolerance” gene ontologies, we found functions related to metabolism (such as carbohydrate metabolism, fructose metabolism, and chitin metabolism). However, we also discovered gene ontologies related to RNA polymerase II transcription, immune response, and regulation of response to reactive oxygen species (Fig. 5a). Within our 125 “resistance” gene ontologies, we found functions related to metabolism (such as carbohydrate metabolism, chitin metabolism, oligosaccharide biosynthesis, and general metabolism) (Fig. 5b).

To visually explore gene expression patterns related to tolerance and resistance, we used hierarchical clustering to separate candidate DEGs into common patterns, and then visualized these clusters using parallel coordinate lines superimposed onto side-by-side boxplots. To reduce overplotting of parallel coordinate lines, we again used hierarchical clustering techniques to separate DEGs into common patterns. Perhaps unsurprisingly, we still see a substantial amount of noise (inconsistency between replicates) in our resulting candidate DEGs (Additional files 15 and 16). However, the broad patterns we expect to see still emerge: For example, based on the contrasts we created to obtain the ‘tolerance” candidate DEGs, we expect them to display larger count values in the “NC” group compared to the “NR” group and larger count values in the “VC” group compared to the “VR” group. Indeed, we see this pattern in the associated parallel coordinate plots (Additional file 15). Likewise, based on the contrasts we created to obtain the ‘resistance” candidate DEGs, we still expect them to display larger count values in the “VC” group compared to the “VR” group, but we no longer expect to see a difference between the “NC” and “NR” groups. We do generally see these expected patterns in the associated parallel coordinate plots: While there are large outliers in the “NC” group, the “NR” replicates are no longer typically below a standardized count of zero (Additional file 16). The genes in Cluster 3 follow the expected pattern the most distinctively (Additional file 16).

### Post hoc analysis

To better understand sources of transcriptomic noise, we explored whether pathogen response measurements (virus titers and mortality), which varied widely across samples, were correlated with observed patterns in gene expression.

The R-squared values between gene read counts and pathogen response measurements were generally low (R-squared <0.1) across our dataset (Additional file 1: Table S9). We further explored whether clusters of DEGs showed higher correlations with pathogen response measurements than non-DEGs (the latter serving as a control, where we do not expect a correlation). A Kruskal–Wallis test was used to determine if R-squared distributions of DEG clusters significantly differed from those in the rest of the data. The *p*-values and Bonferroni correction values for each of the 36 tests (as described in the methods section) is provided in Additional file 1: Table S9. Distribution of the R-squared values for DEG cluster read counts and pathogen response metrics is provided in Additional file 19. An overall trend emerges to suggest that DEGs may have significantly larger correlation with the pathogen response measurements compared to non-DEGs.

## Discussion

Challenges to honey bee health are a growing concern, in particular the combined, interactive effects of nutritional stress and pathogens [12]. In this study, we used RNA-sequencing to probe mechanisms underlying honey bee responses to two effects, diet quality and infection with the prominent virus of concern, IAPV. In general, we found a major nutritional transcriptomic response, with nearly 2000 transcripts changing in response to diet quality (rockrose/poor diet versus chestnut/good diet). The majority of these genes were upregulated in response to high quality diet, and these genes were over-represented for functions such as nutrient signaling metabolism (insulin resistance), immune response (Notch signaling and JaK-STAT pathways), and carbon metabolism (Additional file 1: Table S2). These data suggest high quality nutrition may allow bees to alter their metabolism, favoring investment of energy into immune responses.

One of the few studies that has investigated transcriptomic response to nutrition in honey bees similarly found that pollen upregulates genes related to macromolecule metabolism, insulin pathways, and TOR pathways [48]. Diet effects on transcriptomics have been more extensively studied in the insect model Drosophila. One recent transcriptomic study in *Drosophila melanogaster* reported an overexpression of genes related to immunity, metabolism, and hemocyanin in a high-fat diet and overexpression of genes related to cell cycle activity, DNA binding and transcription, and CHK kinase-like protein activity in a high-sugar diet [54]. This same study also discovered an upregulation of genes related to peptide and carbohydrate processing in both high-fat and high-sugar diets, a finding the authors attributed to a general increase in caloric intake. Another recent study investigated the transcriptomic effects of diets high in protein relative to sugar, diets high in sugar relative to protein, and diets with equal amounts of protein and sugar [55]. *Drosophila mojavensis* and *Drosophila arizonae* showed substantial differential expression between the dietary conditions: genes involved in carbohydrate and lipid metabolism were upregulated in response to high sugar low protein diets and genes involved in juvenile hormone (JH) and ecdysone were upregulated in response to low sugar high protein diets. Interestingly, prior studies have suggested that JH regulates body size by controlling ecdysone production, which modifies insulin signaling [56]. As we saw in our study, these studies generally suggest that diet differences may relate to gene expression changes in metabolism and immune responses in honey bees.

While some insect systems have shown relatively low transcriptional responses to dicistrovirus infection [93, 94], previous work on honey bees has revealed many hundreds of DEGs [49]. Discrepancies between datasets may be due to noise and complexity of the honey bee microbiome. The transcriptomic response to virus infection in our experiment was fairly limited. We found only 43 differentially expressed transcripts, some with known immune functions such as an MD-2 lipid recognition protein that is particularly implicated in the recognition of pathogen products and argonaute-2, a protein that plays a central role in RNA silencing (Table 1). We also found genes related to transcriptional regulation, including Histone H4, Rho guanine nucleotide exchange factor 11, and transcriptional regular Myc-B, which is a regular gene that codes for a transcription factor. We additionally found Tropomyosin-like, a gene involved in muscle contraction. The small number of DEGs in this study may be partly explained by the large amount of noise in the data (Fig. 4 and Additional files 2B, 4, 11, 12, 13, and 14) and baseline viral titers observed in our control bees (Fig. 2).

There have been numerous studies on the transcriptomic effects of virus infection in model organisms like fruit flies and mosquitoes that can provide a useful framework for interpreting virus responses in honey bees. These studies have showed that RNA silencing is a major antiviral strategy, along with transcriptional pausing, Toll pathways, IMD pathways, JAK/STAT pathways, and Toll-7-autophagy pathways [60, 61]. Recent transcriptomic studies in honey bees have shown similar hallmarks of these same antiviral defense mechanisms, including RNA silencing, Toll pathways, IMD pathways, JAK/STAT pathways, autophagy, and endocytosis [62]. It is important to note that general immune responses to viral infection in insects might be an indirect result of cellular damage [61]. In fact, every virus-host interaction has its own particularities derived from the diverse methods of replication and infection cycle evolved by different viruses. An intricate set of pro- and anti-virus host factors such as ribosomal proteins and autophagy pathways are involved, but the response depends on the virus species, as has been elucidated in Drosophila [60, 61]. In addition, a non-sequence-specific antiviral response mediated by unspecific dsRNA pathway was discovered in honey bees [50, 95]. In the case of dicistroviruses, few works have studied the impact of IAPV infection at transcriptional level. Chen et al. 2014 analyzed responses to IAPV infection in larvae and workers using microarrays [51]. Many of the DEGs found were involved in immune response and energy-related metabolism, particularly in adults but not in brood. The authors propose this observed difference could be connected to latent infections in larvae (where host immunity is not perturbed) versus acute infections in adulthood (induced by stressors faced during development) [51]. IAPV acute infection also alters the DNA methylation pattern of numerous genes that do not overlap the genes that are up- or down-regulated at the transcriptional level [49]. These works reiterate the conclusion that viruses trigger particular antiviral mechanisms by different means and depending on several factors. The honey bee antiviral pathways induced by specific viruses were recently reviewed [62]; it is noteworthy that many honey bee factors discovered by transcriptomics need further characterization to uncover their role in controlling (or promoting) viral infection in honey bees.

Given the noisy nature of our data, and our desire to home in on genes with real expression differences, we compared our data to the Galbraith study [49], which also examined bees response to IAPV infection. In contrast to our study, Galbraith et al. identified a large number of virus responsive transcripts, and generally had less noise in their data (Fig. 3 and Additional files 2A, 5, 6, 7, 8, 9, and 10). To identify the most consistent virus-responsive genes from our study, we looked for overlap in the DEGs associated with virus infection on both experiments. We found a large, statistically significant (*p*-value <2.2e-16) overlap, with 26/38 (68%) of virus-responsive DEGs from our study also showing response to virus infection in Galbraith et al. (Fig. 6). This result gives us confidence that, although noisy, we were able to uncover reliable, replicable gene expression responses to virus infection with our data.

Data visualization is a useful method to identify noise and robustness in RNA-sequencing data [79]. In this study, we used extensive data visualization to improve the interpretation of our RNA-sequencing results. For example, the DESeq2 package comes with certain visualization options that are popular in RNA-sequencing analysis. One of these visualization is the principal component analysis (PCA) plot, which allows users to visualize the similarity between samples within a dataset. We could determine from this plot that indeed the Galbraith data may show more similarity between its replicates and differences between its treatments compared to our data (Additional file 2). However, the PCA plot only shows us information at the sample level. We wanted to investigate how these differences in the signal:to:noise ratios of the datasets would affect the structure of any resulting DEGs. As a result, we also used three plotting techniques from the bigPint package: We investigated the 1019 virus-related DEGs from the Galbraith dataset and the 43 virus-related DEGs from our dataset using parallel coordinate lines, scatterplot matrices, and litre plots. To prevent overplotting issues in our graphics, we used a hierarchical clustering technique for the parallel coordinate lines to separate the set of DEGs into smaller groups. We also needed to examine four subsets of samples from our dataset to make effective use of the scatterplot matrices. After these tailorizations, we determined that the same patterns we saw in the PCA plots regarding the entire dataset extended down the pipeline analysis into the DEG calls: Even the DEGs from the Galbraith dataset showed more similarity between their replicates and differences between their treatments compared to those from our data. However, the 365 DEGs from the Galbraith data in Cluster 1 of Fig. 3 showed an inconsistent first replicate in the treatment group (“V.1”), which was something we observed in the PCA plot. This indicates that this feature also extended down the analysis pipeline into DEG calls. Despite the differences in signal between these two datasets, there was substantial overlap in the resulting DEGs. We believe these visualization applications can be useful for future researchers analyzing RNA-sequencing data to quickly and effectively ensure that the DEG calls look reliable or at least overlap with DEG calls from similar studies that look reliable. We also expect this type of visualization exploration can be especially crucial when studying wild populations with high levels of genetic and environmental variation between replicates and/or when using experiments that may lack rigid design control.

One of the goals of this study was to use our RNA-sequencing data to assess whether transcriptomic responses to diet quality and virus infection provide insight into whether high quality diet can buffer bees from pathogen stress via mechanisms of “resistance” or “tolerance”. Recent evidence has suggested that overall immunity is determined by more than just “resistance” (the reduction of pathogen fitness within the host by mechanisms of avoidance and control) [96]. Instead, overall immunity is related to “resistance” in conjunction with “tolerance” (the reduction of adverse effects and disease resulting from pathogens by mechanisms of healing) [96, 97]. Immune-mediated resistance and diet-driven tolerance mechanisms are costly and may compete with each other [97, 98]. Data and models have suggested that selection can favor an optimum combination of both resistance and tolerance [99–102]. We attempted to address this topic through specific gene expression contrasts (Table 2), accompanied by GO analysis of the associated gene lists. We found an approximately equal number of resistance (*n* = 125) and tolerance (*n* = 122) related candidate DEGs, suggesting both processes may be playing significant roles in dietary buffering from pathogen induced mortality. Resistance candidate DEGs had functions related to several forms of metabolism (chitin and carbohydrate), regulation of transcription, and cell adhesion (Fig. 5b). Tolerance candidate DEGs had functions related to carbohydrate metabolism and chitin metabolism; however, they also showed functions related to immune response, including RNA polymerase II transcription (Fig. 5a). Previous studies have shown that transcriptional pausing of RNA polymerase II may be an innate immune response in *D. melanogaster* that allows for a more rapid response by increasing the accessibility of promoter regions of virally induced genes [103]. These possible immunological defense mechanisms within our “tolerance” candidate DEGs and metabolic processes within our “resistance” candidate DEGs may provide additional evidence of feedbacks between diet and disease in honey bees [12]. Thus, our study uses transcriptome data to generate lists of candidate genes that can be the focus of future investigations to better experimentally test putative roles of tolerance and resistance genes in this system.

There were several limitations in this study that could be improved upon in future studies. For instance, our comparison between the Galbraith data (single-drone colonies) and our data (naturally-mated colonies) was limited by numerous extraneous variables between these studies. In addition to different molecular pipelines and bioinformatic preprocessing pipelines used between these studies, the Galbraith study focused on worker honey bees that were fed sugar and artificial pollen diets, whereas we used whole bodies and categorized only into inoculated vs. non-inoculated groups; noise may have been introduced through different responses in asymptomatic bees. Also, Galbraith’s bees were sampled at 24 h while ours were sampled at 36 h. Furthermore, the Galbraith data used eviscerated abdomens with attached fat bodies and observations to determine behaviorally symptomatic bees whereas we used whole bodies and categorized only into inoculated vs. non-inoculated groups. There are also differences in the hours post inoculation and possible differences in the inoculation amount between the studies. Further differences between the studies can be found in their corresponding published methods sections [11, 49]. The different factors between these two studies may be critical because particular antiviral factors in honey bees are linked to specific viruses, specific developmental stages, the analyzed tissue, the route of inoculation, and the time (post-inoculation) during which the study was performed. This was clearly demonstrated when comparing honey bee responses to two related iflaviruses with very different infection dynamics, SBV vs. DWV [52]. Authors observed differences in induction of defensin and hymenoptaecin immune-related genes, and suggested the results reflect adaptations to the different routes of transmission [52].

Moreover, our comparative visualization assessment between these two datasets was also somewhat limited because the virus effect in the Galbraith study used three replicates for each level, whereas the virus effect in our study used twelve replicates for each level that were actually further subdivided into six replicates for each diet level. Hence the apparent reduction in noise observed in the Galbraith data compared to our data in the PCA plots, parallel coordinate plots, scatterplot matrices, and litre plots may be an inadvertent product of the smaller number of replicates used and the lack of a secondary treatment group rather than solely the reduction in genetic variability through the single-drone colony design itself. With this in mind, while our current efforts may be a starting point, future studies can shed more light on signal:to:noise and differential expression differences between naturally-mated colony designs and single-drone colony designs by controlling for extraneous factors more strictly than what we were able to do in the current line of work.

In addition, this study used a whole body RNA-sequencing approach. In future related studies, it may be informative to use tissue-specific methods. Previous work has shown that even though IAPV replication occurs in all honey bee tissues, it localizes more in gut and nerve tissues and in the hypopharyngeal glands. Likewise, the highest IAPV titers have been observed in gut tissues [41]. Recent evidence suggests that RNA-sequencing of composite structures (rather than specific tissues) in honey bees leads to false negatives, implying that genes strongly differentially expressed in particular structures may not reach significance within the composite structure [104]. These studies have also found that within a composite extraction, structures therein may contain opposite patterns of differential expression. If we were to repeat this same experimental design at a more refined tissue level, we would likely provide more detailed answers to our original transcriptomic questions. Another future direction related to this work would be to integrate multiple omics datasets to investigate monofloral diet quality and IAPV infection in honey bees. Indeed, previous studies in honey bees have found that multiple omics datasets do not always align in a clear-cut manner, and hence may broaden our understanding of the molecular mechanisms being explored [49].

## Conclusions

To the best of our knowledge, there are few to no studies investigating honey bee gene expression specifically related to monofloral diets, and few to no studies examining honey bee gene expression related to the combined effects of diet in any general sense and viral inoculation in any general sense. It also remains unknown whether the protective effects of good diet in honey bees is due to direct effects on immune function (resistance) or indirect effects of energy availability on vigor and health (tolerance). We attempted to address these unresolved areas by conducting a two-factor RNA-sequencing study that examined how monofloral diets and IAPV inoculation influence gene expression patterns in honey bees. Overall, our data suggest complex transcriptomic responses to multiple stressors in honey bees. Diet has the capacity for large and profound effects on gene expression and may set up the potential for both resistance and tolerance to viral infection, adding to previous evidence of possible feedbacks between diet and disease in honey bees [12].

Moreover, this study also demonstrated the benefits of using data visualizations and multiple datasets to address inherently messy biological data. For instance, by verifying the substantial overlap in our DEG lists to those obtained in another study that addressed a similar question using specimens with less genetic variability, we were able to place much higher confidence in the differential gene expression results from our otherwise noisy data. We also suggested that comparing results derived from multiple studies varying in level of genetic and environmental variability may allow researchers to identify transcriptomic patterns that are concurrently more realistic and less noisy. Altogether, we hope our results underline the merits of using data visualization techniques and multiple datasets to understand and interpret RNA-sequencing datasets.

## Additional files

Additional file 1 In all tables, “C” represents chestnut diet, “R” represents rockrose diet, “N” represents control non-inoculated, and “V” represents virus-inoculated. **Table 1:** Number of DEGs across three analysis pipelines for the (A) diet main effect in our study, (B) virus main effect in our study, and (C) virus main effect in the Galbraith study. **Table 2:** Pathways related to the 1,033 DEGs upregulated in the chestnut treatment from the diet main effect. **Table 3:** Pathways related to the 881 DEGs upregulated in the rockrose treatment from the diet main effect. **Table 4:** GO analysis results for the 601 DEGs upregulated in the NC treatment from the NC versus NR treatment pair analysis. These DEGs represent genes upregulated in noninoculated honey bees given high quality chestnut pollen versus low quality rockrose pollen. **Table 5:** GO analysis results for the 340 DEGs upregulated in the NR treatment from the NC versus NR treatment pair analysis. These DEGs represent genes upregulated in noninoculated honey bees given low quality rockrose pollen versus high quality chestnut pollen. **Table 6:** GO analysis results for the 247 DEGs upregulated in the VC treatment from the VC versus VR treatment pair analysis. These DEGs
represent genes upregulated in inoculated honey bees given high quality chestnut pollen versus low quality rockrose pollen. **Table 7:** GO analysis results for the 129 DEGs upregulated in the VR treatment from the VC versus VR treatment pair analysis. These DEGs represent genes upregulated in inoculated honey bees given low quality rockrose pollen versus high quality chestnut pollen. **Table 8:** Number of DEGs across three analysis pipelines for all six treatment pair combinati ons between the diet and virus factor. **Table 9:** Kruskal-Wallis p-value and Bonferroni corrections for the 36 combinations of DEG lists, pathogen response metrics, and cluster number. (XLS 28 kb).

Additional file 2 PCA plots for the Galbraith dataset (A) and for our dataset (B). “V” represents virus-inoculated, and “N” represents control non-inoculated. The x-axis represents the principal component with the most variation and the y-axis represents the principal component with the second-most variation. (PNG 250 kb)

Additional file 3 Parallel coordinate plots of the 1914 DEGs after hierarchical clustering of size six between the chestnut and rockrose groups of our study. Here “C” represents chestnut samples, and “R” represents rockrose samples. The vertical red line indicates the distinction between treatment groups. We see from this plot that the DEG designations for this dataset do not appear as clean compared to what we saw in the Galbraith dataset in Fig. 3. (PNG 2031 kb)

Additional file 4 Example litre plots of the nine DEGs with the lowest FDR values from the 43 virus-related DEGs of our dataset. “N” represents non-inoculated control samples and “V” represents virus-treated samples. Most of the magenta points (representing the 144 combinations of samples between treatment groups for a given DEG) do not reflect the expected pattern as clearly compared to what we saw in the litre plots of the Galbraith data. They are not as clustered together (representing replicate inconsistency) and they sometimes cross the *x=y* line (representing lack of difference between treatment groups). This finding reflects what we saw in the messy looking parallel coordinate lines of Fig. 4. (PNG 1160 kb)

Additional file 5 Example litre plots of the nine DEGs with the lowest FDR values from the 365 DEGs in Cluster 1 (originally shown in Fig. 3) of the Galbraith dataset. “N” represents non-inoculated control samples and “V” represents virus-treated samples. Most of the light orange points (representing the nine combinations of samples between treatment groups for a given DEG) deviate from the *x=y* line in a tight bundle as expected. (PNG 964 kb)

Additional file 6 Example litre plots of the nine DEGs with the lowest FDR values from the 327 DEGs in Cluster 2 (originally shown in Fig. 3) of the Galbraith dataset. “N” represents non-inoculated control samples and “V” represents virus-treated samples. Most of the dark orange points (representing the nine combinations of samples between treatment groups for a given DEG) deviate from the *x=y* line in a compact clump as expected. However, they are not as tightly bunched together compared to what we saw in the example litre plots of Cluster 1 (shown in Additional file 5). As a result, what we see in these litre plots reflects what we saw in the parallel coordinate lines of Fig. 3: The replicate consistency in the Cluster 1 DEGs is not as clean as that in the Cluster 2 DEGs, but is still relatively clean. (PNG 1018 kb)

Additional file 7 The 365 DEGs from the first cluster of the Galbraith dataset (originally shown in Fig. 3) superimposed as light orange dots onto all genes as black dots in the form of a scatterplot matrix. The data has been standardized. “N” represents non-inoculated control samples and “V” represents virus-treated samples. We confirm that the DEGs mostly follow the expected structure, with their placement deviating from the *x=y* line in the treatment scatterplots, but adhering to the *x=y* line in the replicate scatterplots. However, we do see that sample “V.1” may be somewhat inconsistent in these DEGs, as its presence in the replicate scatterplots
shows DEGs deviating from the *x=y* line more than expected and its presence in the treatment scatterplots shows DEGs adhering to the *x=y* line more than expected. This inconsistent sample was something we observed in Fig. 3. (PNG 562 kb)

Additional file 8 The 327 DEGs from the second cluster of the Galbraith dataset (originally shown in Fig. 3) superimposed as dark orange dots onto all genes as black dots in the form of a scatterplot matrix. The data has been standardized. “N” represents non-inoculated control samples and “V” represents virus-treated samples. We confirm that the DEGs mostly follow the expected structure, with their placement deviating from the *x=y* line in the treatment scatterplots, but adhering to the *x=y* line in the replicate scatterplots. (PNG 589 kb)

Additional file 9 The 224 DEGs from the third cluster of the Galbraith dataset (originally shown in Fig. 3) superimposed as turquoise dots onto all genes as black dots in the form of a scatterplot matrix. The data has been standardized. “N” represents non-inoculated control samples and “V” represents virus-treated samples. We confirm that the DEGs mostly follow the expected structure, with their placement deviating from the *x=y* line in the treatment scatterplots, but adhering to the *x=y* line in the replicate scatterplots. (PNG 618 kb)

Additional file 10 The 103 DEGs from the fourth cluster of the Galbraith dataset (originally shown in Fig. 3) superimposed as pink dots onto all genes as black dots in the form of a scatterplot matrix. The data has been standardized. “N” represents non-inoculated control samples and “V” represents virus-treated samples. We confirm that the DEGs mostly follow the expected structure, with their placement deviating from the *x=y* line in the treatment scatterplots, but adhering to the *x=y* line in the replicate scatterplots. We also see that the second replicate from the virus-treated sample (“V.2”) may be somewhat inconsistent in these DEGs, as its presence in the replicate scatterplots results in the DEGs unexpectedly deviating from the *x=y* line and its presence in the treatment scatterplots results in the DEGs unexpectedly adhering to the *x=y* line. This inconsistent sample was something we observed in Fig. 3. (PNG 560 kb)

Additional file 11 The 43 virus-related DEGs from our dataset superimposed as magenta dots onto all genes in the form of a scatterplot matrix. Only replicates 1, 2, and 3 are shown from both treatment groups. The data has been standardized. “N” represents non-inoculated control samples and “V” represents virus-treated samples. We see that, compared to the scatterplot matrices from certain clusters of the Galbraith data, the 43 DEGs from this subset of six samples from our data do not paint as clear of a picture, sometimes unexpectedly deviating from the *x=y* line in the replicate plots and sometimes unexpectedly adhering to the *x=y* line in the treatment plots. (PNG 584 kb)

Additional file 12 The 43 virus-related DEGs from our dataset superimposed as magenta dots onto all genes in the form of a scatterplot matrix. Only replicates 4, 5, and 6 are shown from both treatment groups. The data has been standardized. “N” represents non-inoculated control samples and “V” represents virus-treated samples. We see that, compared to the scatterplot matrices from certain clusters of the Galbraith data, the 43 DEGs from this subset of six samples from our data do not paint as clear of a picture, and most of them unexpectedly adhere to the *x=y* line in the treatment plots. (PNG 579 kb)

Additional file 13 The 43 virus-related DEGs from our dataset superimposed as magenta dots onto all genes in the form of a scatterplot matrix. Only replicates 7, 8, and 9 are shown from both treatment groups. The data has been standardized. “N” represents non-inoculated control samples and “V” represents virus-treated samples. We see that, compared to the scatterplot matrices
from certain clusters of the Galbraith data, the 43 DEGs from this subset of six samples from our data do not paint as clear of a picture, sometimes unexpectedly deviating from the *x=y* line in the replicate plots and sometimes unexpectedly adhering to the *x=y* line in the treatment plots. (PNG 565 kb)

Additional file 14 The 43 virus-related DEGs from our dataset superimposed onto all genes in the form of a scatterplot matrix. Only replicates 10, 11, and 12 are shown from both treatment groups. The data has been standardized. “N” represents non-inoculated control samples and “V” represents virus-treated samples. We see that, compared to the scatterplot matrices from certain clusters of the Galbraith data, the 43 DEGs from this subset of six samples from our data do not paint as clear of a picture, and most of them unexpectedly deviate from the *x=y* line in the virus-related replicate plots. (PNG 587 kb)

Additional file 15 Parallel coordinate plots of the 122 DEGs after hierarchical clustering of size four between the “tolerance” candidate DEGs. Here “N” represents non-inoculated control group, “V” represents treatment of virus, “C” represents high quality chestnut diet, and “R” represents low quality rockrose diet. The vertical red line indicates the distinction between treatment groups. We see there is considerable noise in the data (non-consistent replicate values), but that the general patterns of the DEGs follow what we expect based on our “tolerance” contrast. (PNG 1741 kb)

Additional file 16 Parallel coordinate plots of the 125 DEGs after hierarchical clustering of size four between the “resistance” candidate DEGs. Here “N” represents non-inoculated control group, “V” represents treatment of virus, “C” represents high quality chestnut diet, and “R” represents low quality rockrose diet. The vertical red line indicates the distinction between treatment groups. We see there is considerable noise in the data (non-consistent replicate values), but that the general patterns of the DEGs follow what we expect based on our “resistance” contrasts. (PNG 2014 kb)

Additional file 17 Venn diagrams comparing the virus-related DEG overlaps of the Galbraith data from the DESeq2 bioinformatics pipelines used in the Galbraith study (labeled as “G.O.”) and the DESeq2 bioinformatics pipelines used in our study (labeled as “G.R”). While we were not able to fully replicate the DEG list published in the Galbraith study, our DEG list maintained significant overlaps with their DEG list. From left to right: Total virus-related DEGs (subplot A), virus-upregulated DEGs (subplot B), control-upregulated DEGs (subplot C). (PNG 164 kb)

Additional file 18 Venn diagrams comparing DEG overlaps across DESeq2, edgeR, and limma for our diet main effect (top row), our virus main effect (middle row), and the Galbraith virus main effect (bottom row). Within a given subplot, “D” represents DESeq2, “E” represents edgeR, and “L” represents limma. From left to right on top row: Total diet-related DEGs (subplot A), chestnut-upregulated DEGs (subplot B), rockrose-upregulated DEGs (subplot C). From left to right on middle row: Total virus-related DEGs (subplot D), virus-upregulated DEGs (subplot E), control-upregulated DEGs in our data (subplot F). From left to right on bottom row: Total virus-related DEGs (subplot G), virus-upregulated DEGs (subplot H), control-upregulated DEGs in the Galbraith data (subplot I) (PNG). With the exception of the limma pipeline resulting in zero DEGs in our virus main effect analysis, we found significant overlaps between DEG lists across the different pipelines (DESeq2, edgeR, and limma). In general, DESeq2 resulted in the largest number of DEGs and limma resulted in the least number of DEGs. (PNG 537 kb)

Additional file 19 Distribution of R-squared values for DEG cluster read counts and pathogen response metrics. Columns left to right: SBV titers, mortality rates, and IAPV
titers. Rows top to bottom: Tolerance candidate DEGs, resistance candidate DEGs, and virus-related DEGs. Each subplot includes five boxplots which represent the R-squared value distributions for four DEG clusters and all remaining non-DEGs in the data. The top number above each boxplot represents the number of genes included. The first four boxplots also include a bottom number, which represents the Kruskal–Wallis *p*-value of the comparison of the R-squared distribution of the cluster and the R-squared distribution of the non-DEG data. (PNG 323 kb)

Additional file 20 **Table 1:** IDs of 1914 DEGs in our diet main effect. **Table 2:** IDs of 43 DEGs in our virus main effect. **Table 3:** IDs of 178 DEGs in our NR versus VR contrast. **Table 4:** IDs of 376 DEGs in our VC versus VR contrast. **Table 5:** IDs of 774 DEGs in our NC versus VR contrast. **Table 6:** IDs of 955 DEGs in our VC versus NR contrast. **Table 7:** IDs of 941 DEGs in our NC versus NR contrast. **Table 8:** IDs of 125 resistance candidate genes. **Table 9:** IDs of 122 tolerance candidate genes. (XLS 1376 kb)

## Abbreviations

DEG: Differentially expressed gene
DWV: Deformed wing virus
GO: Gene ontology
IAPV: Israeli acute paralysis virus
NC: Non-inoculation, high quality pollen (chestnut) pollen treatment
NR: Non-inoculation, low quality (rockrose) pollen treatment
PCD: Principal component analysis
REML: Restricted maximum likelihood
SBV: Sacbrood virus
VC: IAPV-inoculated, high quality pollen (chestnut) pollen treatment
VR: IAPV-inoculated, low quality (rockrose) pollen treatment
