# Supplementary figures and images for: Transcriptomic responses to diet quality and viral infection in Apis mellifera
Source: BMC Genomics. 2019 May 22;20:412. doi: 10.1186/s12864-019-5767-1 (PMC6532243; doi:10.1186/s12864-019-5767-1)

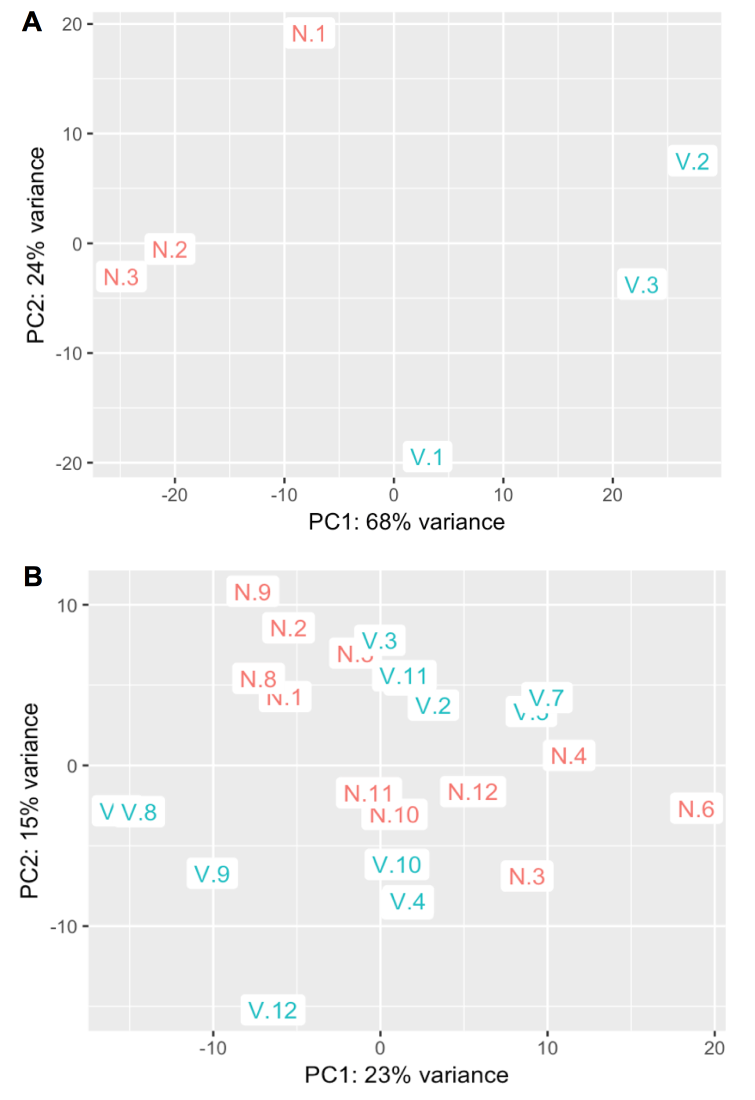

Supplement: Supplementary file 2 — PCA plots for the Galbraith dataset (A) and for our dataset (B). “V” represents virus-inoculated, and “N” represents control non-inoculated. The x-axis represents the principal component with the most variation and the y-axis represents the principal component with the second-most variation. (PNG 250 kb) [file 12864_2019_5767_MOESM2_ESM.png]

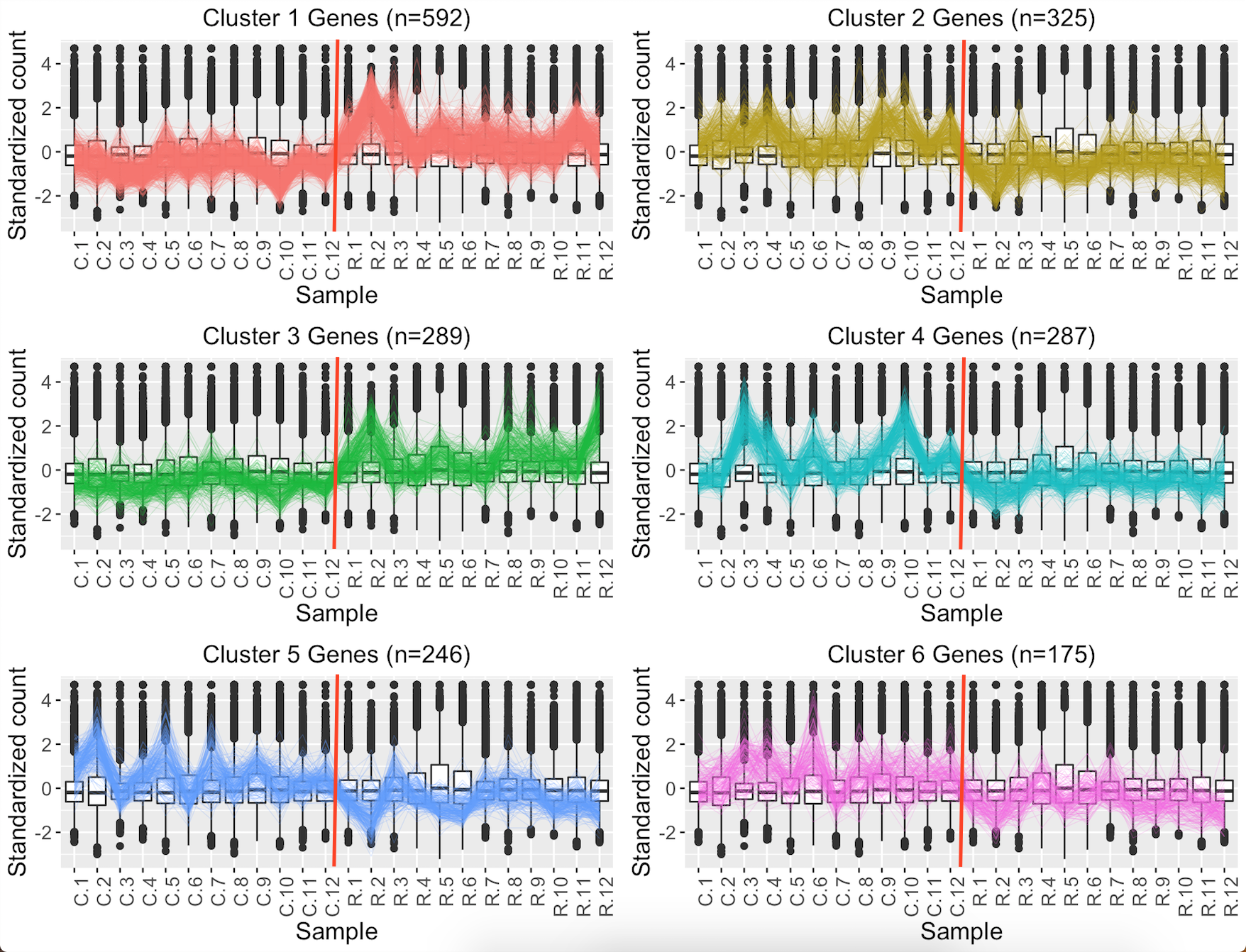

Supplement: Supplementary file 3 — Parallel coordinate plots of the 1914 DEGs after hierarchical clustering of size six between the chestnut and rockrose groups of our study. Here “C” represents chestnut samples, and “R” represents rockrose samples. The vertical red line indicates the distinction between treatment groups. We see from this plot that the DEG designations for this dataset do not appear as clean compared to what we saw in the Galbraith dataset in Fig. 3. (PNG 2031 kb) [file 12864_2019_5767_MOESM3_ESM.png]

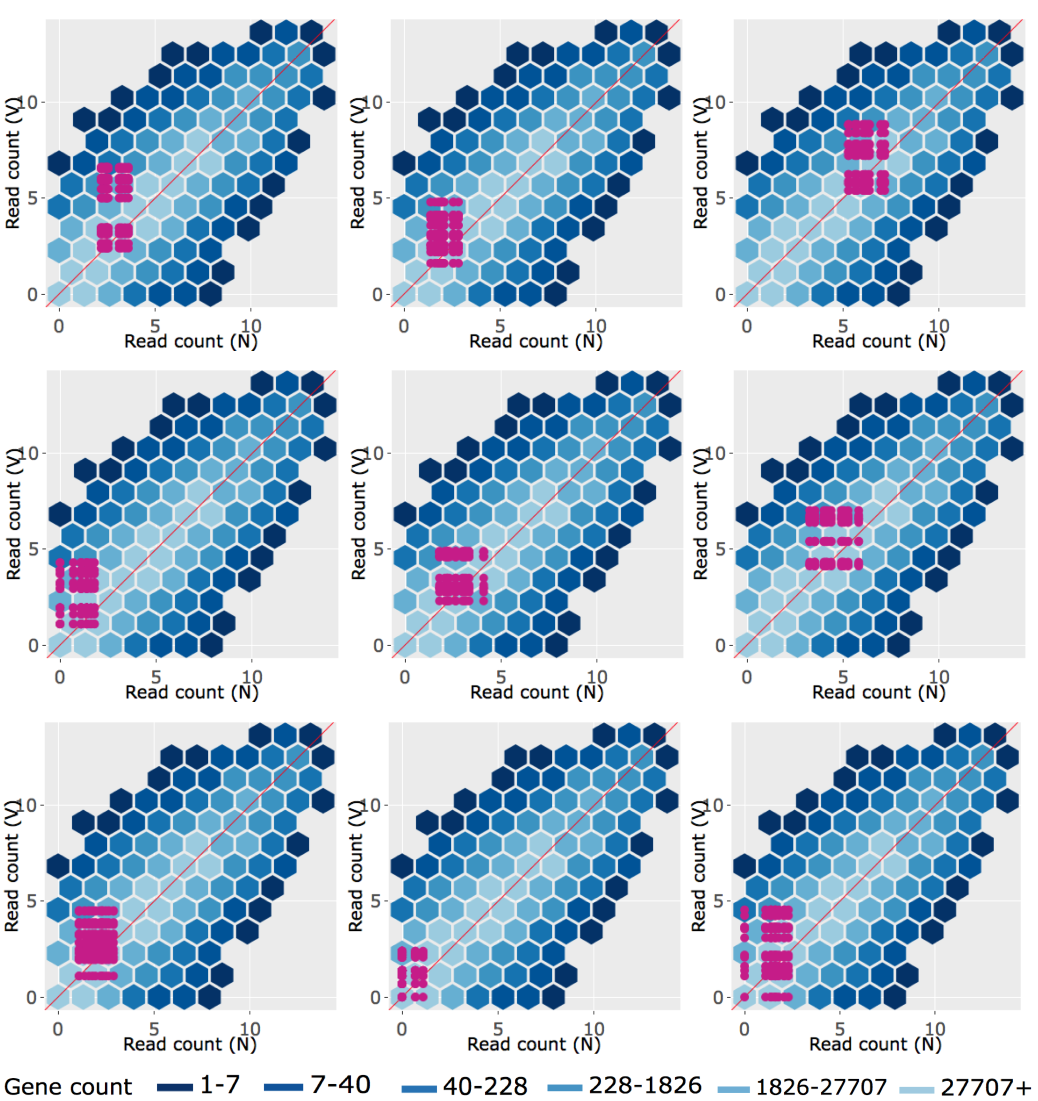

Supplement: Supplementary file 4 — Example litre plots of the nine DEGs with the lowest FDR values from the 43 virus-related DEGs of our dataset. “N” represents non-inoculated control samples and “V” represents virus-treated samples. Most of the magenta points (representing the 144 combinations of samples between treatment groups for a given DEG) do not reflect the expected pattern as clearly compared to what we saw in the litre plots of the Galbraith data. They are not as clustered together (representing replicate inconsistency) and they sometimes cross the x=y line (representing lack of difference between treatment groups). This finding reflects what we saw in the messy looking parallel coordinate lines of Fig. 4. (PNG 1160 kb) [file 12864_2019_5767_MOESM4_ESM.png]

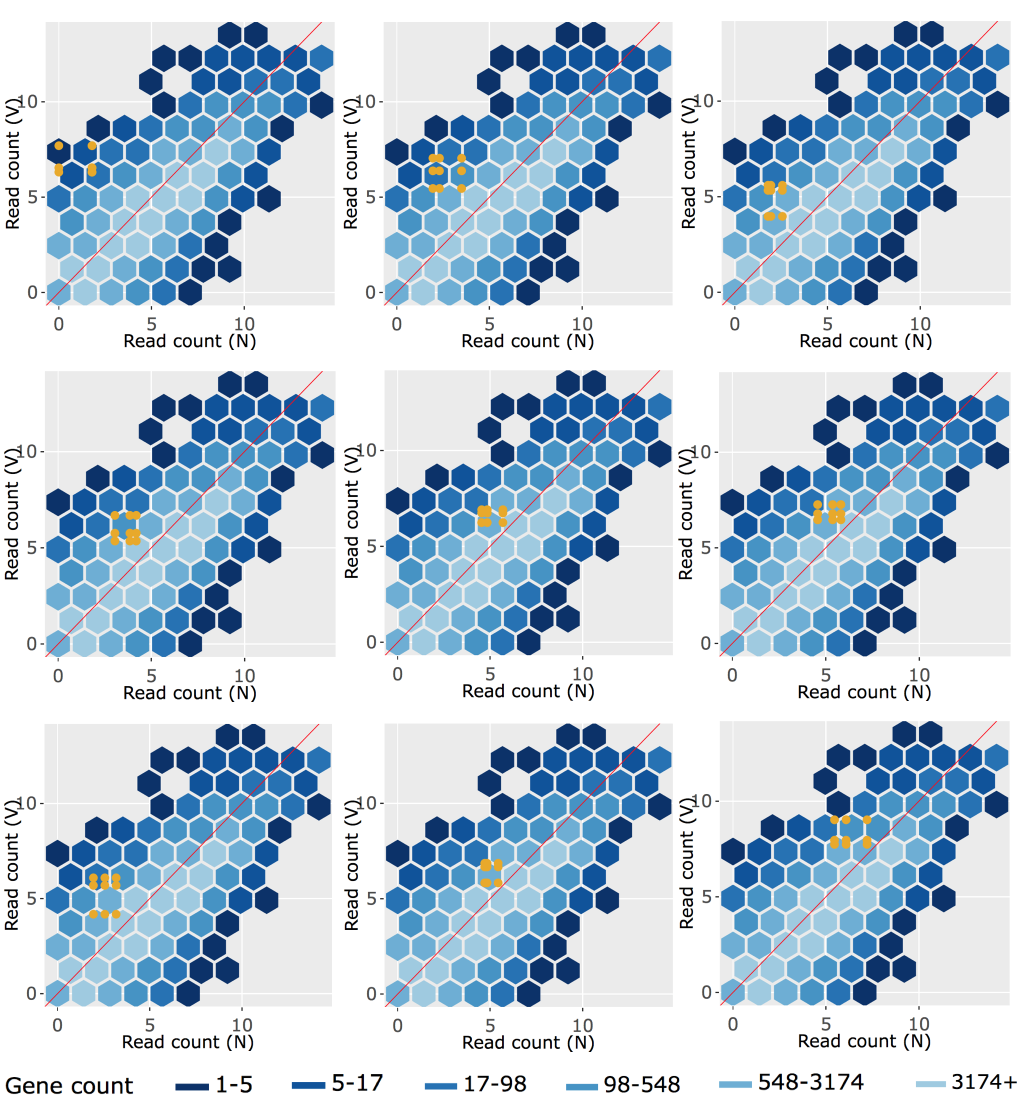

Supplement: Supplementary file 5 — Example litre plots of the nine DEGs with the lowest FDR values from the 365 DEGs in Cluster 1 (originally shown in Fig. 3) of the Galbraith dataset. “N” represents non-inoculated control samples and “V” represents virus-treated samples. Most of the light orange points (representing the nine combinations of samples between treatment groups for a given DEG) deviate from the x=y line in a tight bundle as expected. (PNG 964 kb) [file 12864_2019_5767_MOESM5_ESM.png]

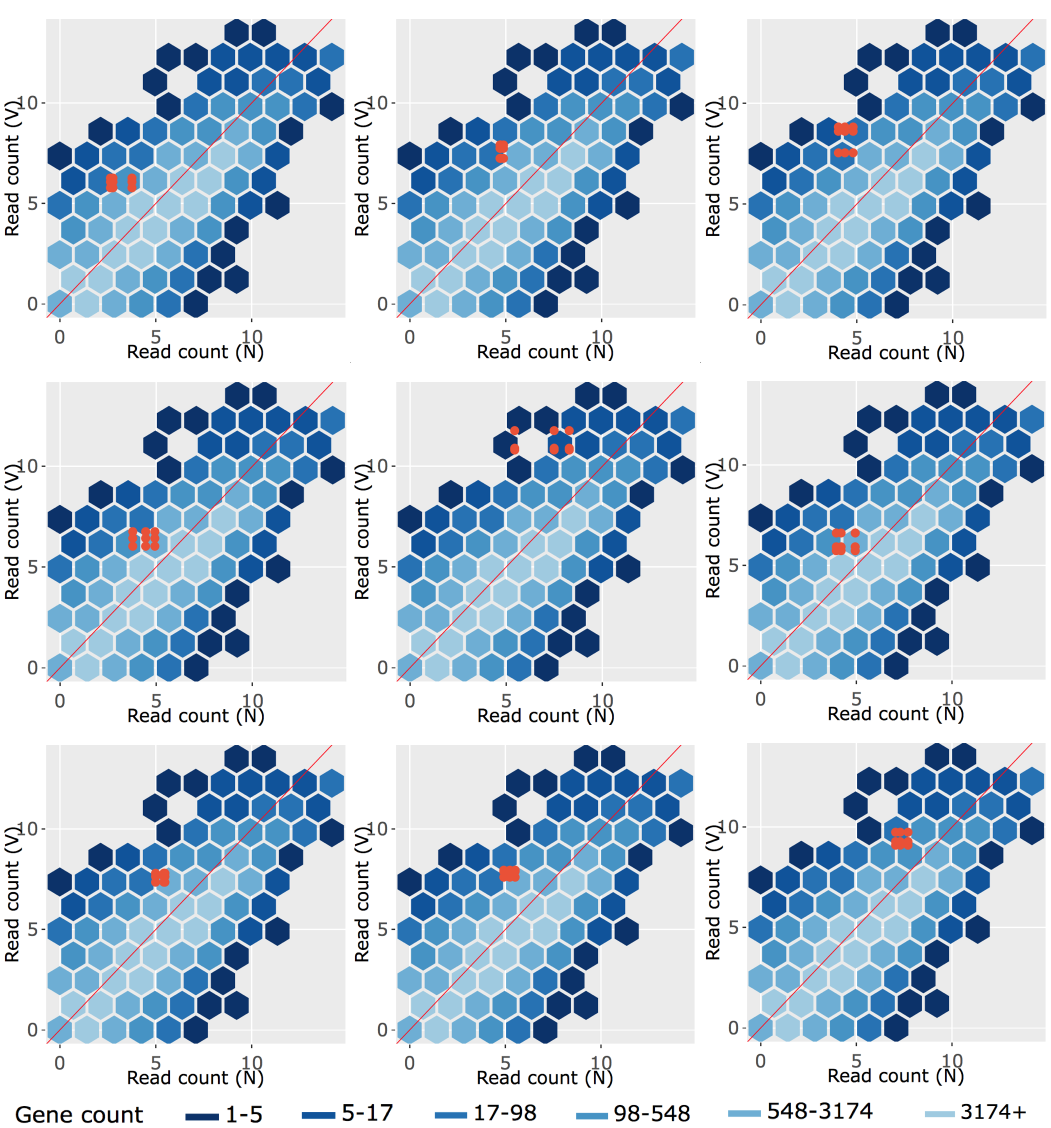

Supplement: Supplementary file 6 — Example litre plots of the nine DEGs with the lowest FDR values from the 327 DEGs in Cluster 2 (originally shown in Fig. 3) of the Galbraith dataset. “N” represents non-inoculated control samples and “V” represents virus-treated samples. Most of the dark orange points (representing the nine combinations of samples between treatment groups for a given DEG) deviate from the x=y line in a compact clump as expected. However, they are not as tightly bunched together compared to what we saw in the example litre plots of Cluster 1 (shown in Additional file 5). As a result, what we see in these litre plots reflects what we saw in the parallel coordinate lines of Fig. 3: The replicate consistency in the Cluster 1 DEGs is not as clean as that in the Cluster 2 DEGs, but is still relatively clean. (PNG 1018 kb) [file 12864_2019_5767_MOESM6_ESM.png]

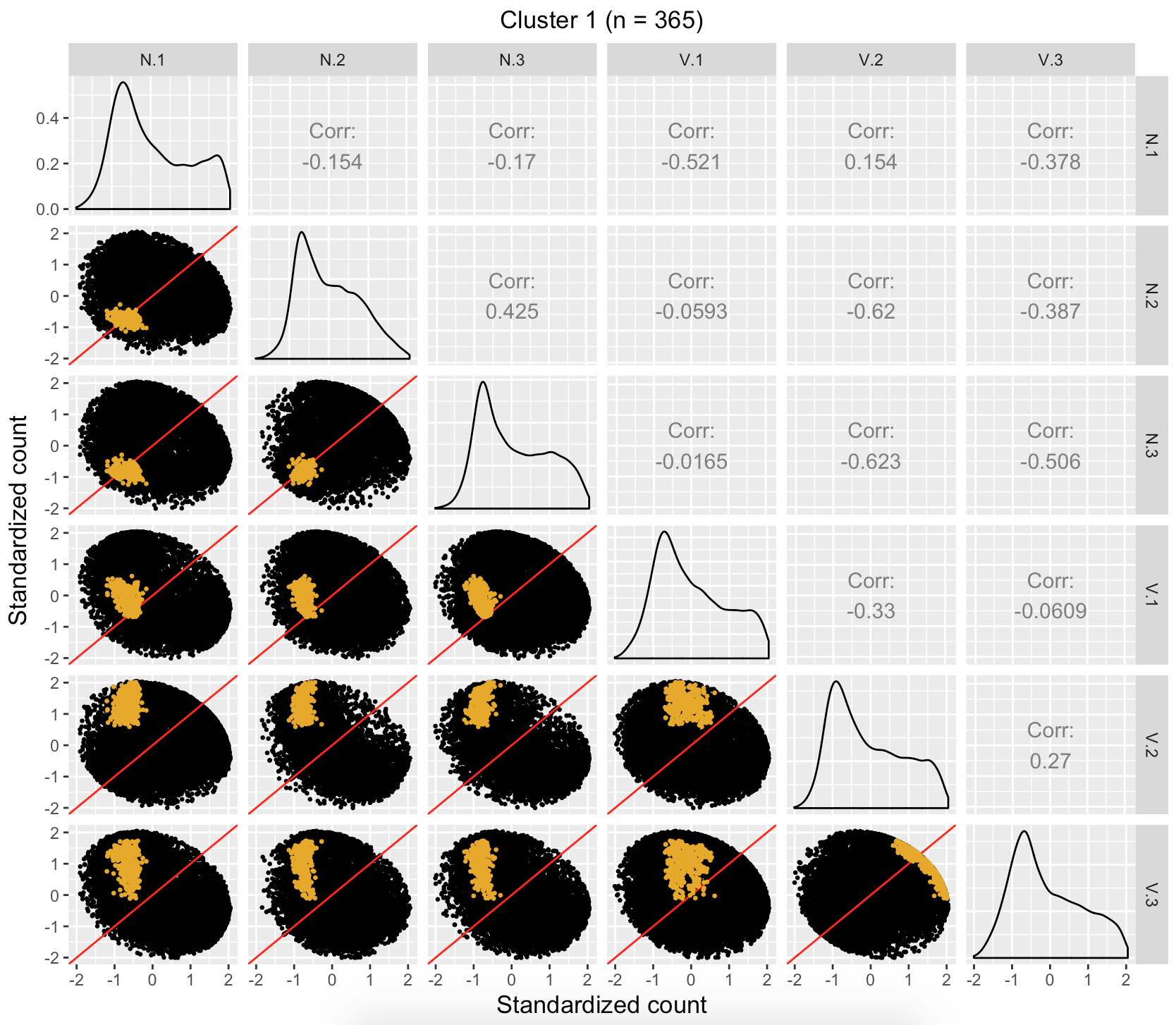

Supplement: Supplementary file 7 — The 365 DEGs from the first cluster of the Galbraith dataset (originally shown in Fig. 3) superimposed as light orange dots onto all genes as black dots in the form of a scatterplot matrix. The data has been standardized. “N” represents non-inoculated control samples and “V” represents virus-treated samples. We confirm that the DEGs mostly follow the expected structure, with their placement deviating from the x=y line in the treatment scatterplots, but adhering to the x=y line in the replicate scatterplots. However, we do see that sample “V.1” may be somewhat inconsistent in these DEGs, as its presence in the replicate scatterplots shows DEGs deviating from the x=y line more than expected and its presence in the treatment scatterplots shows DEGs adhering to the x=y line more than expected. This inconsistent sample was something we observed in Fig. 3. (PNG 562 kb) [file 12864_2019_5767_MOESM7_ESM.png]

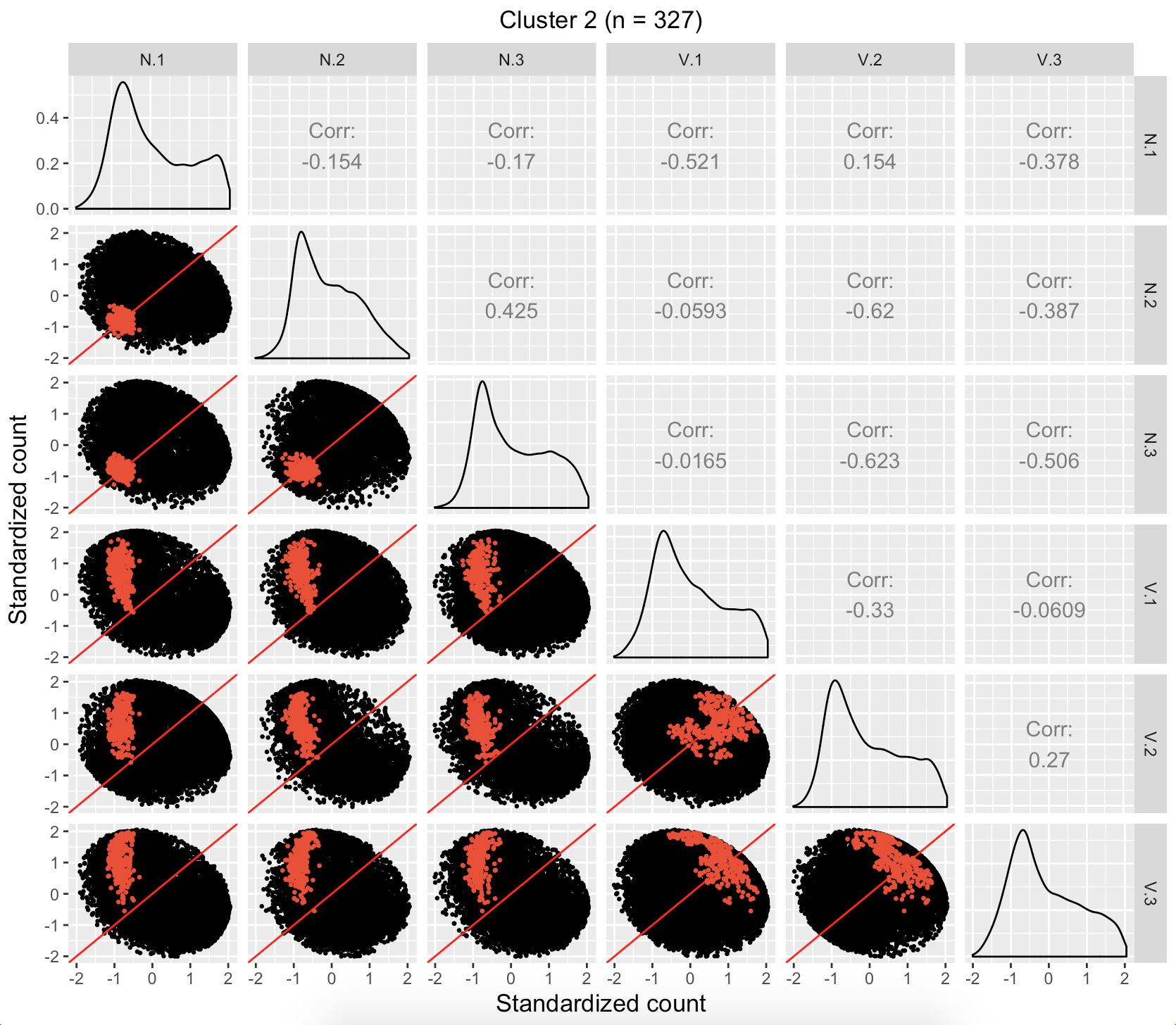

Supplement: Supplementary file 8 — The 327 DEGs from the second cluster of the Galbraith dataset (originally shown in Fig. 3) superimposed as dark orange dots onto all genes as black dots in the form of a scatterplot matrix. The data has been standardized. “N” represents non-inoculated control samples and “V” represents virus-treated samples. We confirm that the DEGs mostly follow the expected structure, with their placement deviating from the x=y line in the treatment scatterplots, but adhering to the x=y line in the replicate scatterplots. (PNG 589 kb) [file 12864_2019_5767_MOESM8_ESM.png]

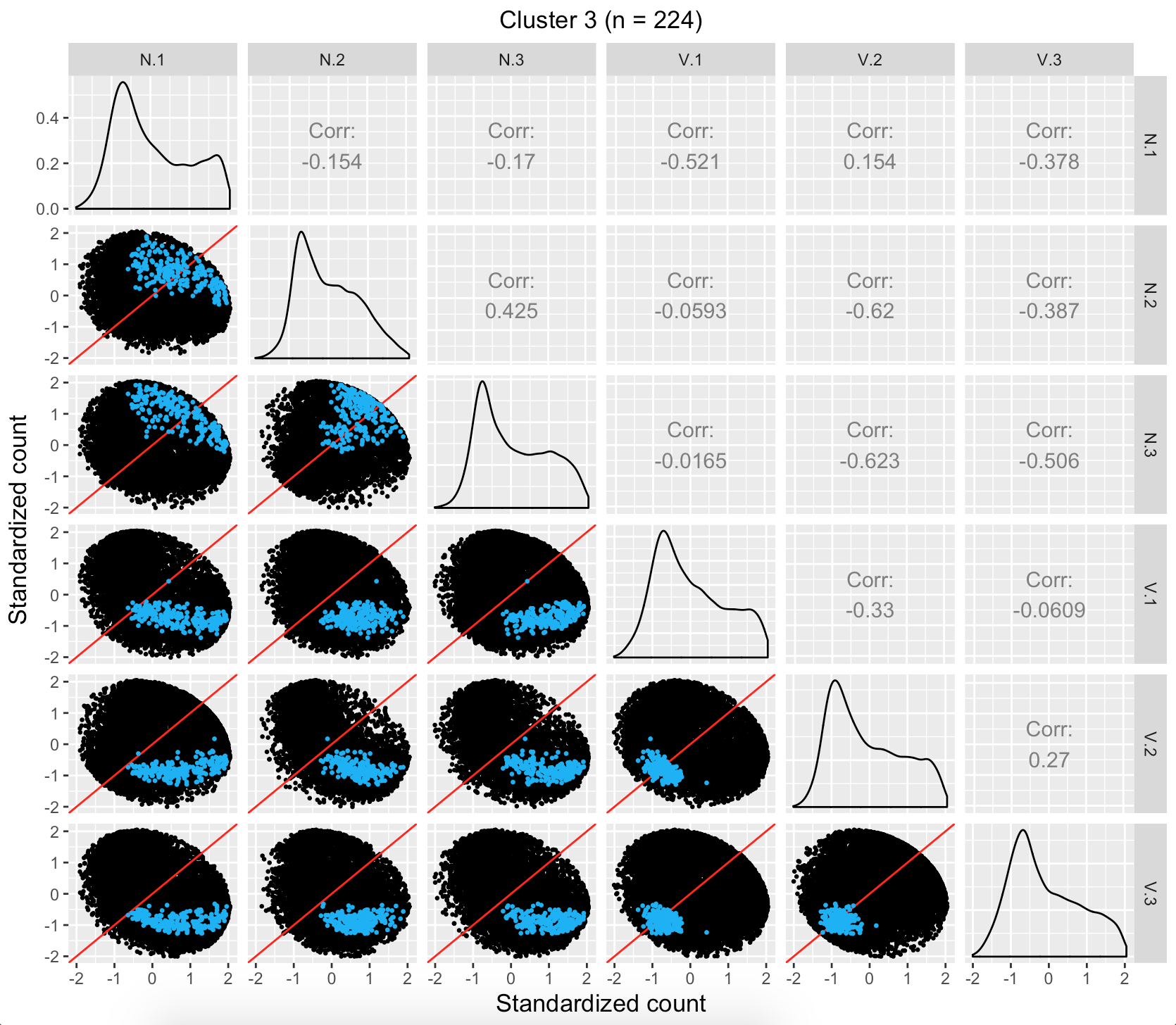

Supplement: Supplementary file 9 — The 224 DEGs from the third cluster of the Galbraith dataset (originally shown in Fig. 3) superimposed as turquoise dots onto all genes as black dots in the form of a scatterplot matrix. The data has been standardized. “N” represents non-inoculated control samples and “V” represents virus-treated samples. We confirm that the DEGs mostly follow the expected structure, with their placement deviating from the x=y line in the treatment scatterplots, but adhering to the x=y line in the replicate scatterplots. (PNG 618 kb) [file 12864_2019_5767_MOESM9_ESM.png]

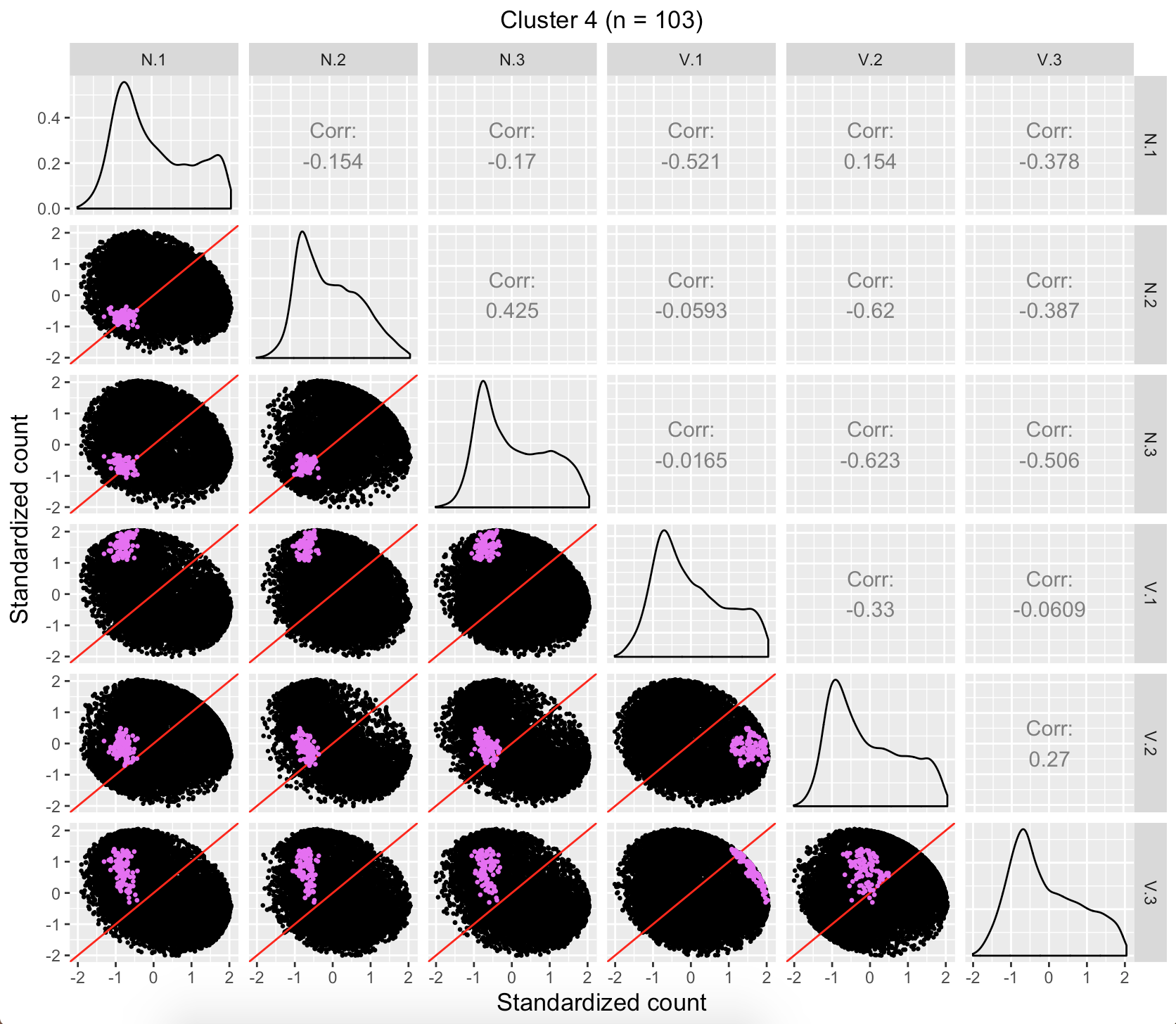

Supplement: Supplementary file 10 — The 103 DEGs from the fourth cluster of the Galbraith dataset (originally shown in Fig. 3) superimposed as pink dots onto all genes as black dots in the form of a scatterplot matrix. The data has been standardized. “N” represents non-inoculated control samples and “V” represents virus-treated samples. We confirm that the DEGs mostly follow the expected structure, with their placement deviating from the x=y line in the treatment scatterplots, but adhering to the x=y line in the replicate scatterplots. We also see that the second replicate from the virus-treated sample (“V.2”) may be somewhat inconsistent in these DEGs, as its presence in the replicate scatterplots results in the DEGs unexpectedly deviating from the x=y line and its presence in the treatment scatterplots results in the DEGs unexpectedly adhering to the x=y line. This inconsistent sample was something we observed in Fig. 3. (PNG 560 kb) [file 12864_2019_5767_MOESM10_ESM.png]

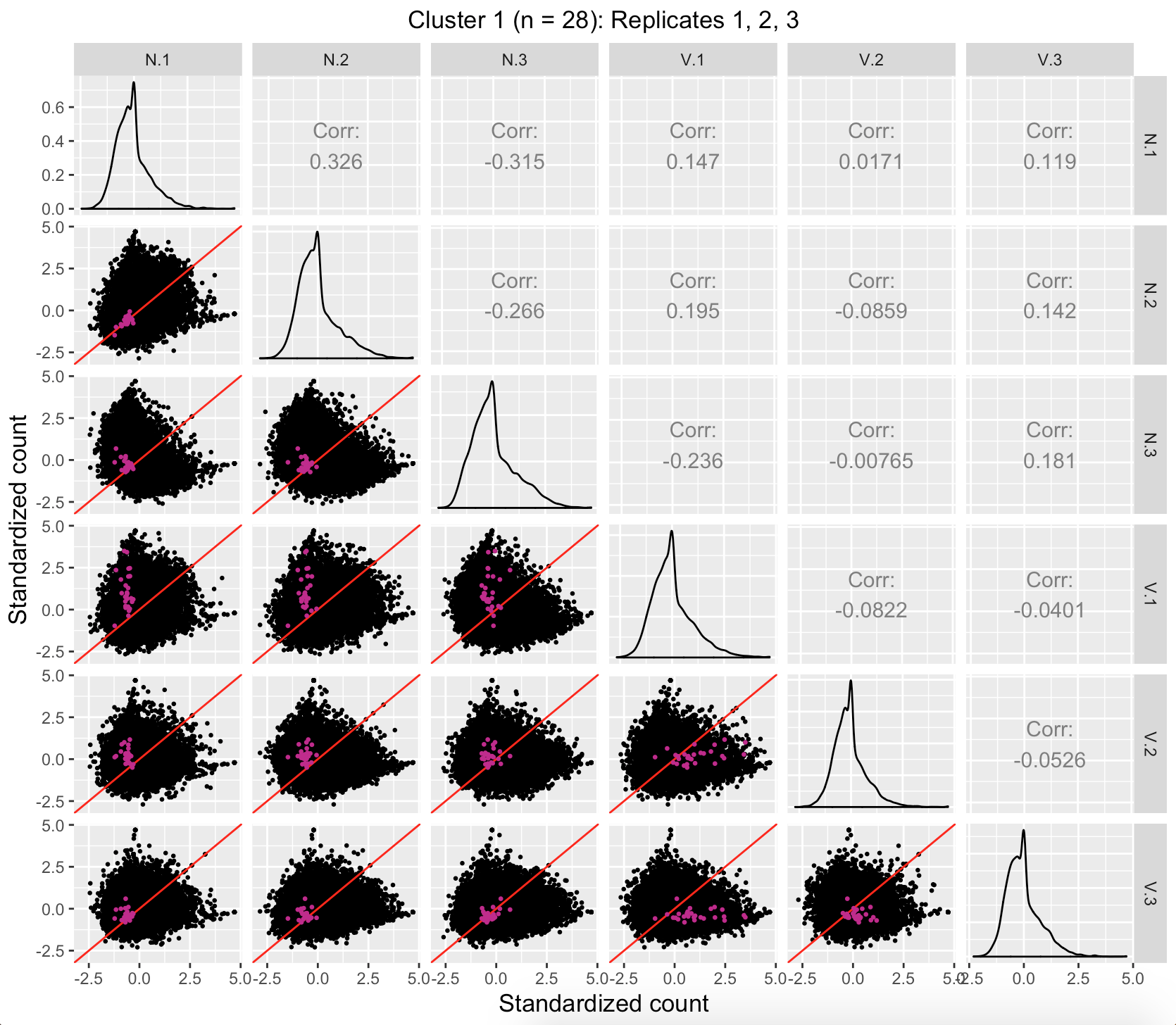

Supplement: Supplementary file 11 — The 43 virus-related DEGs from our dataset superimposed as magenta dots onto all genes in the form of a scatterplot matrix. Only replicates 1, 2, and 3 are shown from both treatment groups. The data has been standardized. “N” represents non-inoculated control samples and “V” represents virus-treated samples. We see that, compared to the scatterplot matrices from certain clusters of the Galbraith data, the 43 DEGs from this subset of six samples from our data do not paint as clear of a picture, sometimes unexpectedly deviating from the x=y line in the replicate plots and sometimes unexpectedly adhering to the x=y line in the treatment plots. (PNG 584 kb) [file 12864_2019_5767_MOESM11_ESM.png]

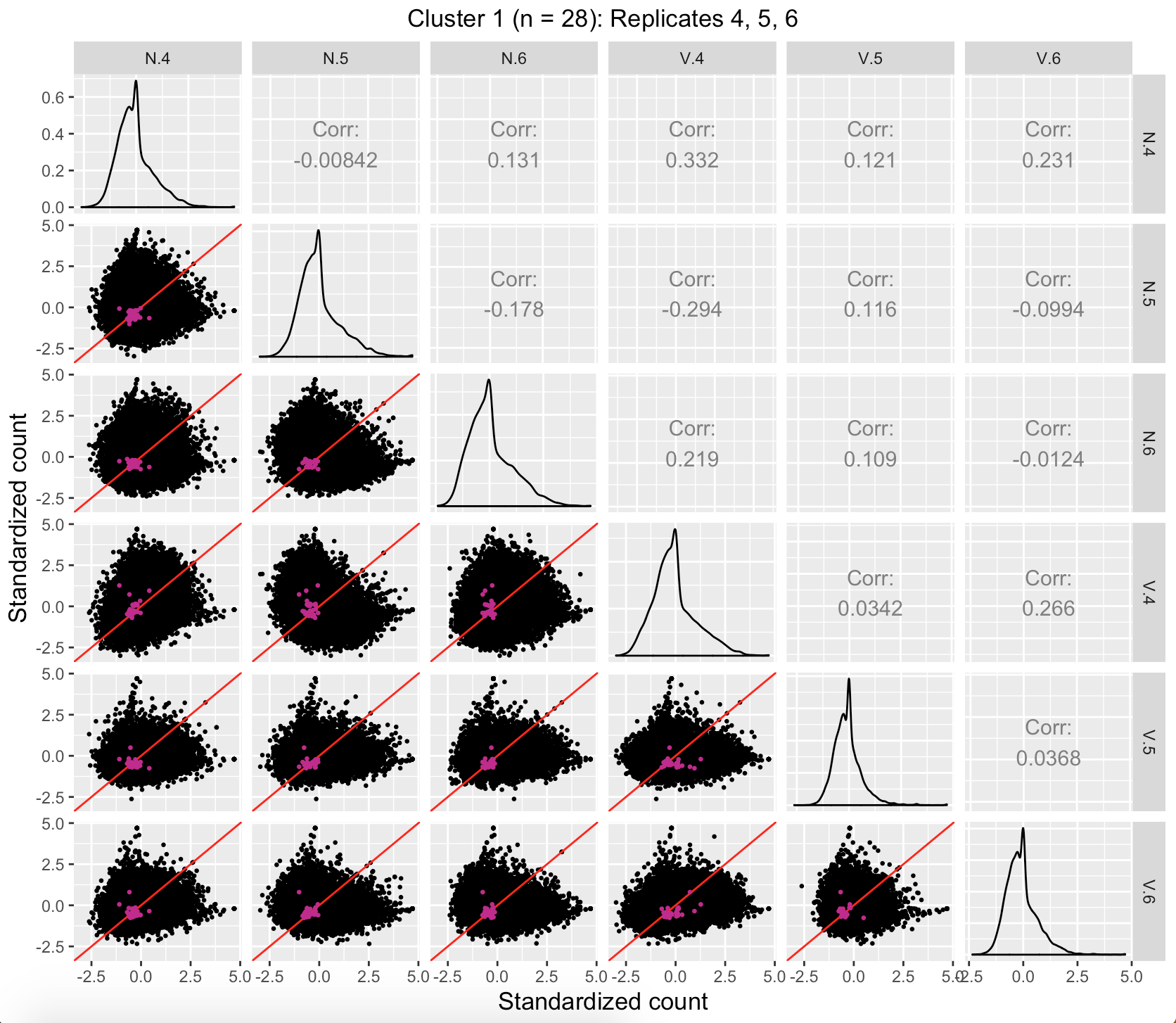

Supplement: Supplementary file 12 — The 43 virus-related DEGs from our dataset superimposed as magenta dots onto all genes in the form of a scatterplot matrix. Only replicates 4, 5, and 6 are shown from both treatment groups. The data has been standardized. “N” represents non-inoculated control samples and “V” represents virus-treated samples. We see that, compared to the scatterplot matrices from certain clusters of the Galbraith data, the 43 DEGs from this subset of six samples from our data do not paint as clear of a picture, and most of them unexpectedly adhere to the x=y line in the treatment plots. (PNG 579 kb) [file 12864_2019_5767_MOESM12_ESM.png]

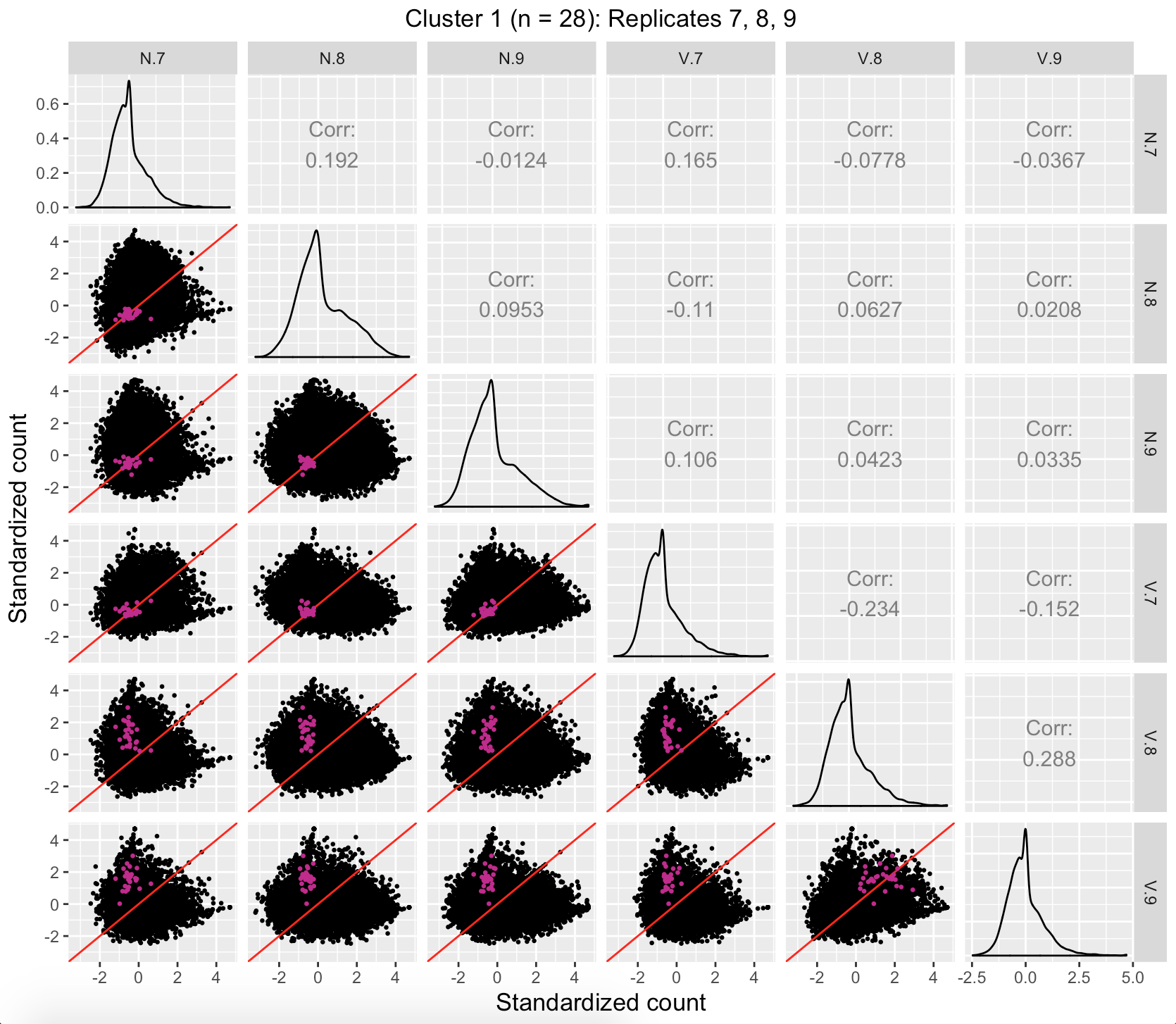

Supplement: Supplementary file 13 — The 43 virus-related DEGs from our dataset superimposed as magenta dots onto all genes in the form of a scatterplot matrix. Only replicates 7, 8, and 9 are shown from both treatment groups. The data has been standardized. “N” represents non-inoculated control samples and “V” represents virus-treated samples. We see that, compared to the scatterplot matrices from certain clusters of the Galbraith data, the 43 DEGs from this subset of six samples from our data do not paint as clear of a picture, sometimes unexpectedly deviating from the x=y line in the replicate plots and sometimes unexpectedly adhering to the x=y line in the treatment plots. (PNG 565 kb) [file 12864_2019_5767_MOESM13_ESM.png]

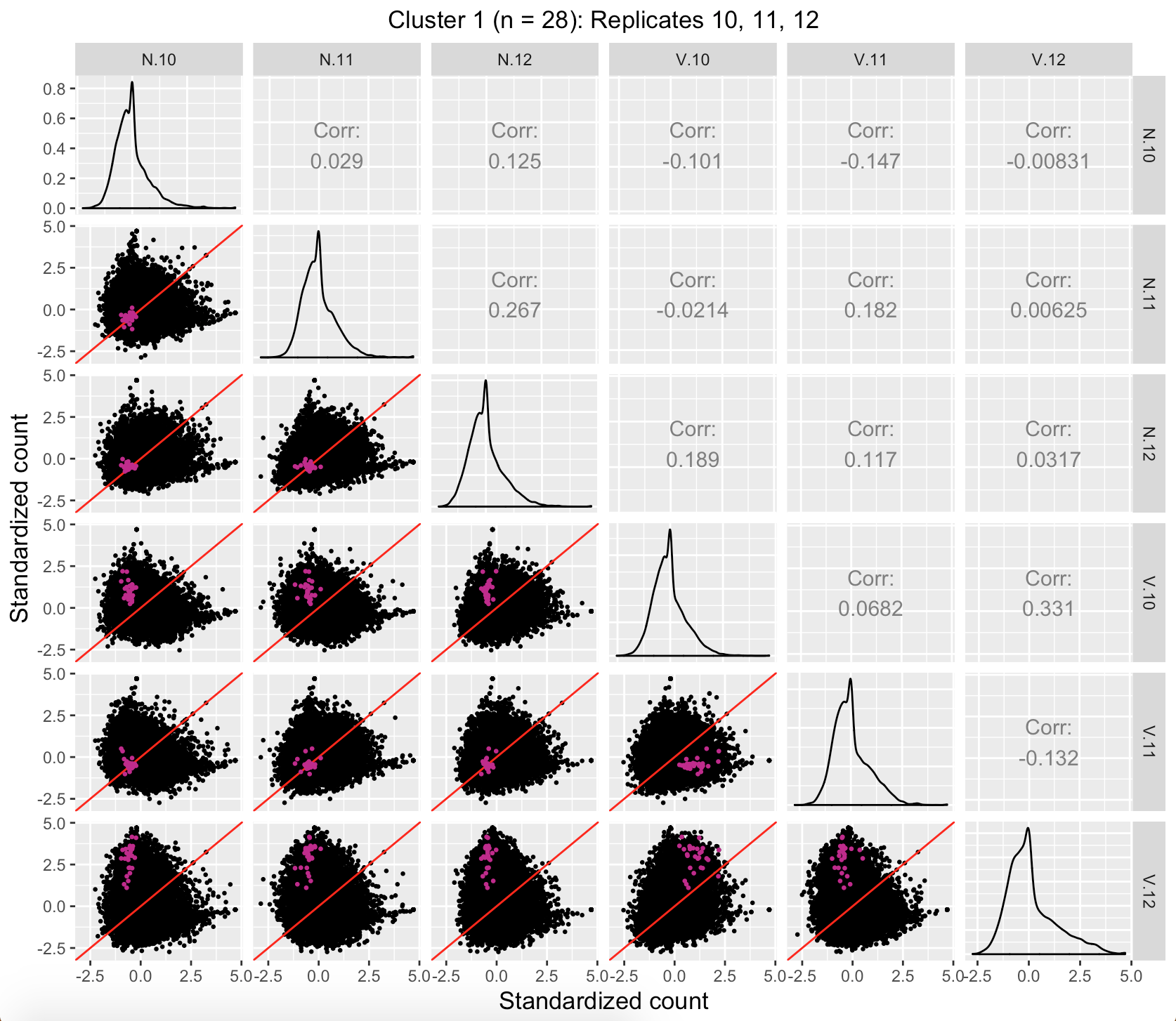

Supplement: Supplementary file 14 — The 43 virus-related DEGs from our dataset superimposed onto all genes in the form of a scatterplot matrix. Only replicates 10, 11, and 12 are shown from both treatment groups. The data has been standardized. “N” represents non-inoculated control samples and “V” represents virus-treated samples. We see that, compared to the scatterplot matrices from certain clusters of the Galbraith data, the 43 DEGs from this subset of six samples from our data do not paint as clear of a picture, and most of them unexpectedly deviate from the x=y line in the virus-related replicate plots. (PNG 587 kb) [file 12864_2019_5767_MOESM14_ESM.png]

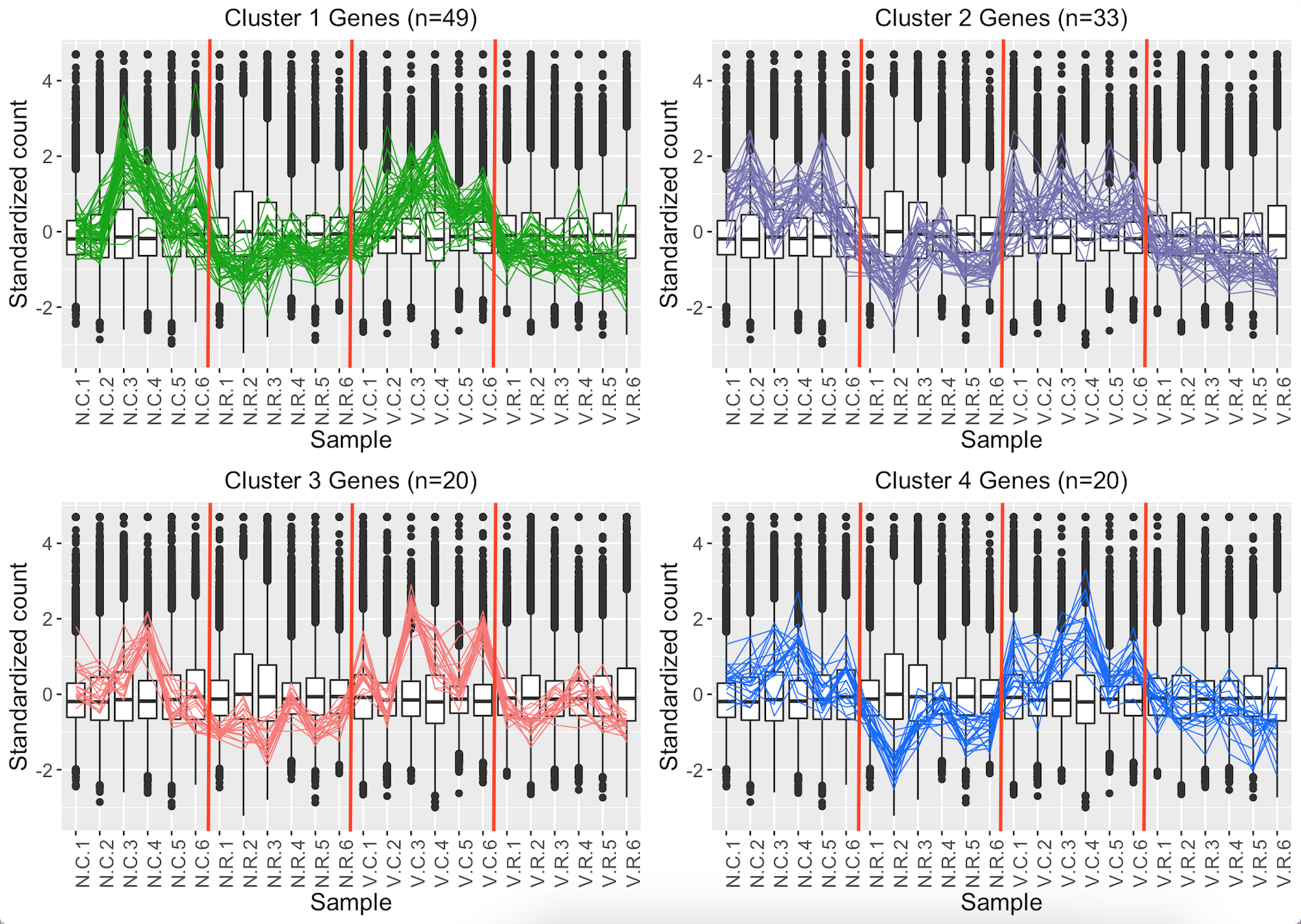

Supplement: Supplementary file 15 — Parallel coordinate plots of the 122 DEGs after hierarchical clustering of size four between the “tolerance” candidate DEGs. Here “N” represents non-inoculated control group, “V” represents treatment of virus, “C” represents high quality chestnut diet, and “R” represents low quality rockrose diet. The vertical red line indicates the distinction between treatment groups. We see there is considerable noise in the data (non-consistent replicate values), but that the general patterns of the DEGs follow what we expect based on our “tolerance” contrast. (PNG 1741 kb) [file 12864_2019_5767_MOESM15_ESM.png]

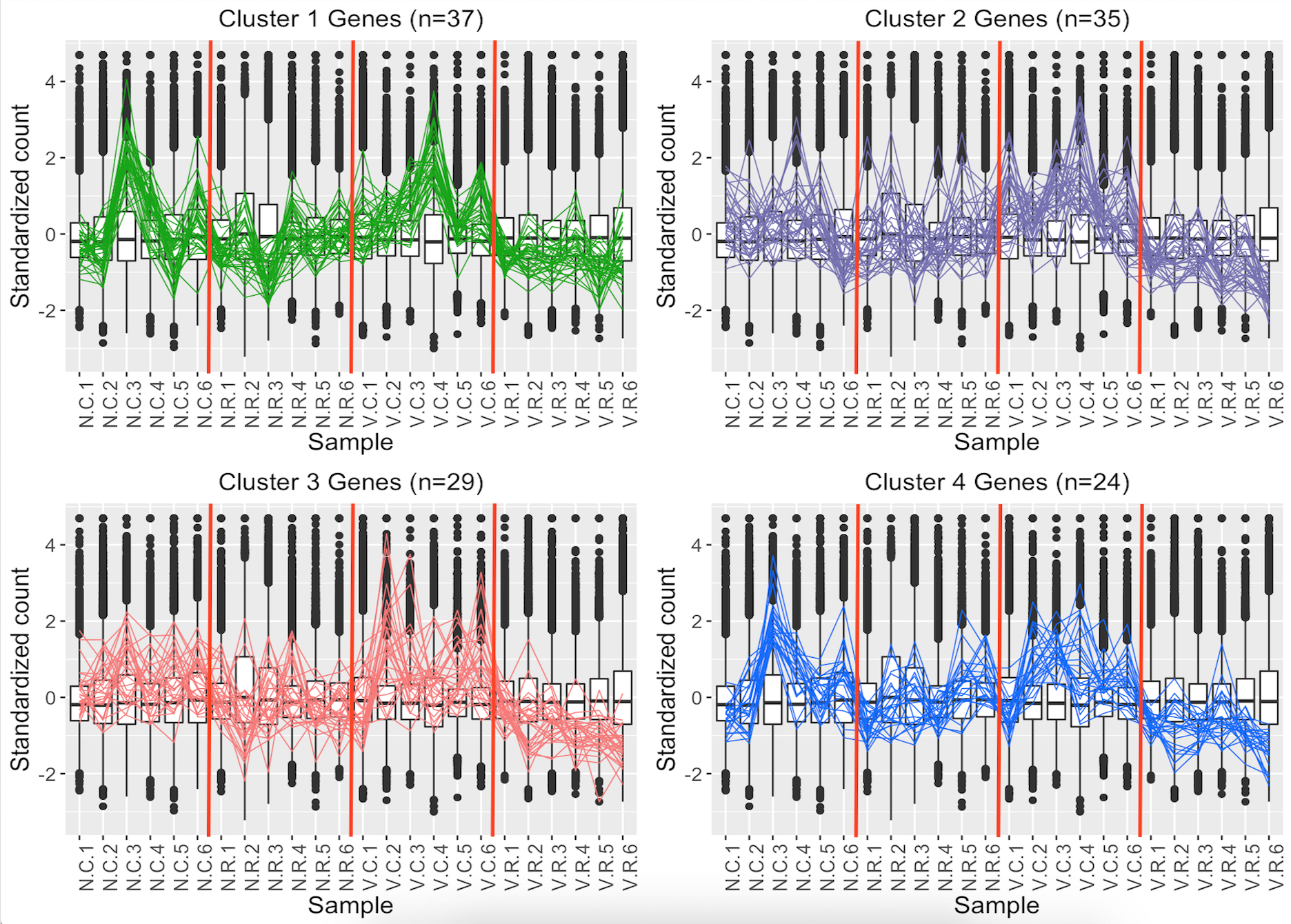

Supplement: Supplementary file 16 — Parallel coordinate plots of the 125 DEGs after hierarchical clustering of size four between the “resistance” candidate DEGs. Here “N” represents non-inoculated control group, “V” represents treatment of virus, “C” represents high quality chestnut diet, and “R” represents low quality rockrose diet. The vertical red line indicates the distinction between treatment groups. We see there is considerable noise in the data (non-consistent replicate values), but that the general patterns of the DEGs follow what we expect based on our “resistance” contrasts. (PNG 2014 kb) [file 12864_2019_5767_MOESM16_ESM.png]

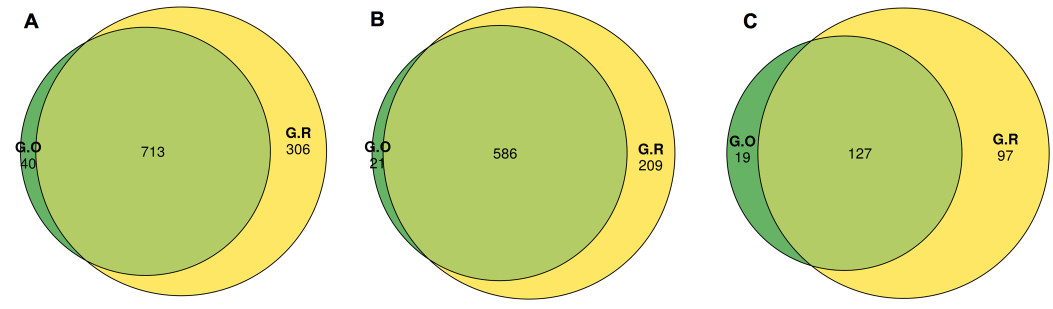

Supplement: Supplementary file 17 — Venn diagrams comparing the virus-related DEG overlaps of the Galbraith data from the DESeq2 bioinformatics pipelines used in the Galbraith study (labeled as “G.O.”) and the DESeq2 bioinformatics pipelines used in our study (labeled as “G.R”). While we were not able to fully replicate the DEG list published in the Galbraith study, our DEG list maintained significant overlaps with their DEG list. From left to right: Total virus-related DEGs (subplot A), virus-upregulated DEGs (subplot B), control-upregulated DEGs (subplot C). (PNG 164 kb) [file 12864_2019_5767_MOESM17_ESM.png]

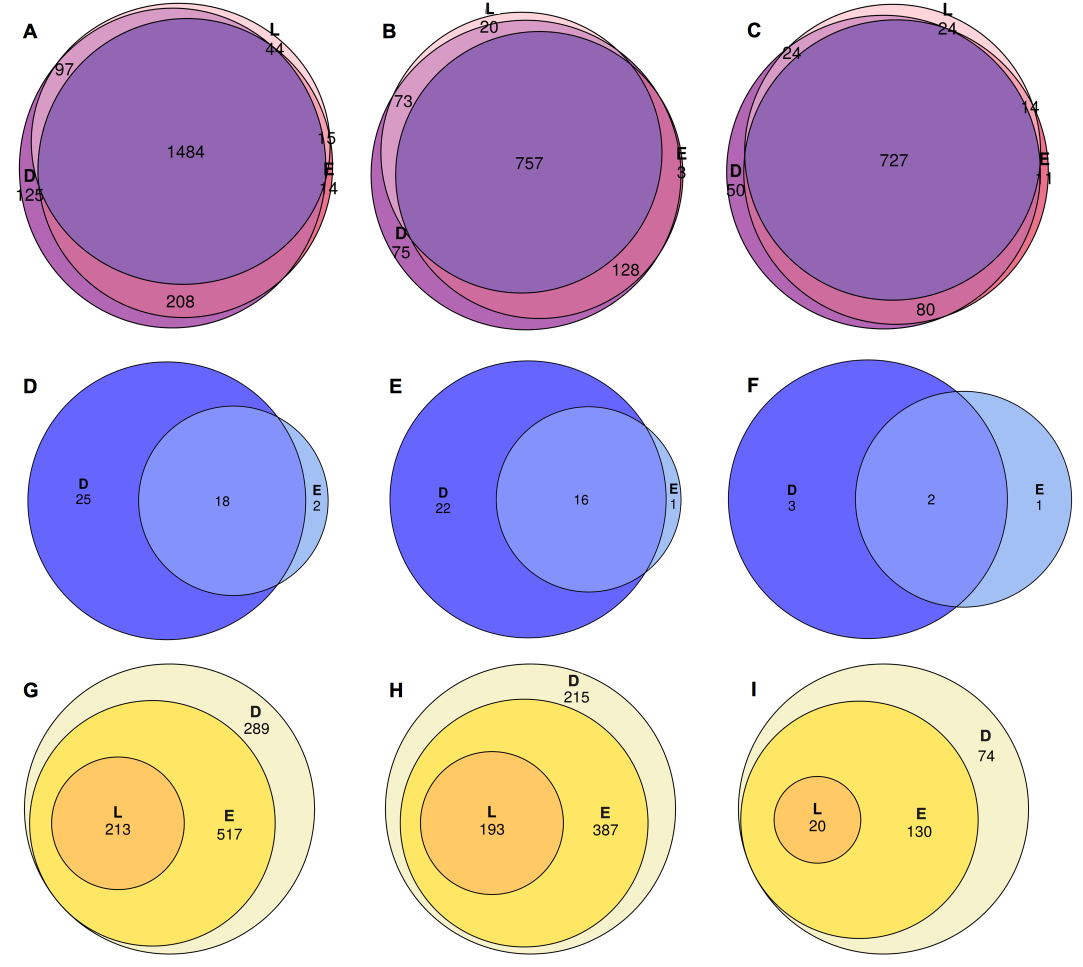

Supplement: Supplementary file 18 — Venn diagrams comparing DEG overlaps across DESeq2, edgeR, and limma for our diet main effect (top row), our virus main effect (middle row), and the Galbraith virus main effect (bottom row). Within a given subplot, “D” represents DESeq2, “E” represents edgeR, and “L” represents limma. From left to right on top row: Total diet-related DEGs (subplot A), chestnut-upregulated DEGs (subplot B), rockrose-upregulated DEGs (subplot C). From left to right on middle row: Total virus-related DEGs (subplot D), virus-upregulated DEGs (subplot E), control-upregulated DEGs in our data (subplot F). From left to right on bottom row: Total virus-related DEGs (subplot G), virus-upregulated DEGs (subplot H), control-upregulated DEGs in the Galbraith data (subplot I) (PNG). With the exception of the limma pipeline resulting in zero DEGs in our virus main effect analysis, we found significant overlaps between DEG lists across the different pipelines (DESeq2, edgeR, and limma). In general, DESeq2 resulted in the largest number of DEGs and limma resulted in the least number of DEGs. (PNG 537 kb) [file 12864_2019_5767_MOESM18_ESM.png]

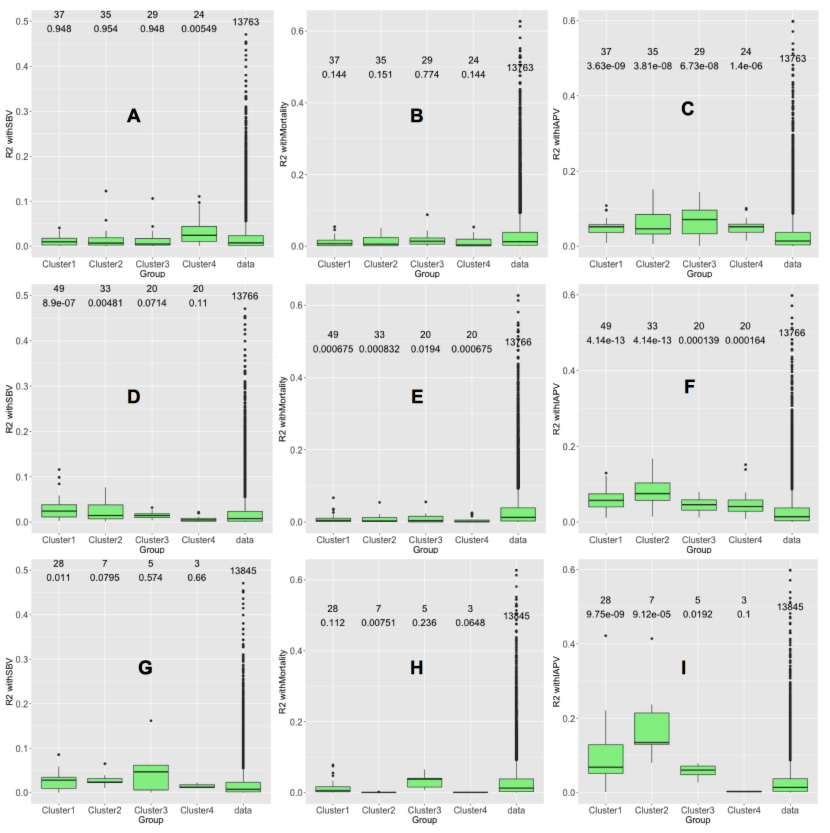

Supplement: Supplementary file 19 — Distribution of R-squared values for DEG cluster read counts and pathogen response metrics. Columns left to right: SBV titers, mortality rates, and IAPV titers. Rows top to bottom: Tolerance candidate DEGs, resistance candidate DEGs, and virus-related DEGs. Each subplot includes five boxplots which represent the R-squared value distributions for four DEG clusters and all remaining non-DEGs in the data. The top number above each boxplot represents the number of genes included. The first four boxplots also include a bottom number, which represents the Kruskal–Wallis p-value of the comparison of the R-squared distribution of the cluster and the R-squared distribution of the non-DEG data. (PNG 323 kb) [file 12864_2019_5767_MOESM19_ESM.png]
